# Supplementary material for: A two-arm parallel-group individually randomised prison pilot study of a male remand alcohol intervention for self-efficacy enhancement: the APPRAISE study protocol
Source: BMJ Open. 2021 Apr 2;11(4):e040636. doi: 10.1136/bmjopen-2020-040636 (PMC8023744; doi:10.1136/bmjopen-2020-040636)
Supplement: Author's manuscript [file bmjopen-2020-040636.draft_revisions.pdf]

BMJ Open is committed to open peer review. As part of this commitment we make the peer review history of every article we publish publicly available.

When an article is published we post the peer reviewers' comments and the authors' responses online. We also post the versions of the paper that were used during peer review. These are the versions that the peer review comments apply to.

The versions of the paper that follow are the versions that were submitted during the peer review process. They are not the versions of record or the final published versions. They should not be cited or distributed as the published version of this manuscript.

BMJ Open is an open access journal and the full, final, typeset and author-corrected version of record of the manuscript is available on our site with no access controls, subscription charges or pay-per-view fees (http://bmjopen.bmj.com).

If you have any questions on BMJ Open's open peer review process please email info.bmjopen@bmj.com

BMJ Open

A two-arm parallel group individually randomised Prison Pilot study of a male Remand Alcohol Intervention for Self-efficacy Enhancement: The APPRAISE Study Protocol

Journal:	*BMJ Open*
Manuscript ID	bmjopen-2020-040636
Article Type:	Protocol
Date Submitted by the Author:	18-May-2020

Complete List of Authors:	Holloway, Aisha; University of Edinburgh, School of Health in Social Sciences<br>Guthrie, Victoria; University of Edinburgh, School of Health in Social Sciences<br>Waller, Gillian; Teesside University<br>Smith, Jamie; University of Edinburgh, School of Health in Social Sciences; Charite Universitatsmedizin Berlin<br>Boyd, Joanne; Teesside University; County Durham Drug and Alcohol Recovery Service, Centre for Change<br>Mercado, Sharon; University of Edinburgh, School of Health in Social Sciences<br>Smith, Pam; University of Edinburgh, The School of Health in Social Science<br>Stenhouse, Rosie; University of Edinburgh, School of Health in Social Sciences<br>Sheikh, Aziz; University of Edinburgh, Division of Community Health Sciences<br>Parker, Richard; University of Edinburgh, Edinburgh Clinical Trials Unit<br>Stoddart, Andrew; The University Of Edinburgh, Edinburgh Health Services Research Unit<br>Conaglen, Philip; NHS Lothian<br>Coulton, Simon; University of Kent, Centre for Health Service Studies<br>Stadler, Gertraud; Charite Universitatsmedizin Berlin; University of Aberdeen<br>Hunt, Kate; University of Stirling, Institute of Social Marketing<br>Bray, Jeremy; University of North Carolina at Greensboro<br>Ferguson, Jennifer; Teesside University<br>Sondhi, Arun; Therapeutic Solutions,<br>Lynch, Kieran; Public Health England<br>Rees, Jessica; University of Edinburgh, Edinburgh Clinical Trials Unit<br>Newbury-Birch, Dorothy; Teesside University,

Keywords:	PUBLIC HEALTH, Substance misuse < PSYCHIATRY, HEALTH ECONOMICS

SCHOLARONE™
Manuscripts

I, the Submitting Author has the right to grant and does grant on behalf of all authors of the Work (as defined in the below author licence), an exclusive licence and/or a non-exclusive licence for contributions from authors who are: i) UK Crown employees; ii) where BMJ has agreed a CC-BY licence shall apply, and/or iii) in accordance with the terms applicable for US Federal Government officers or employees acting as part of their official duties; on a worldwide, perpetual, irrevocable, royalty-free basis to BMJ Publishing Group Ltd ("BMJ") its licensees and where the relevant Journal is co-owned by BMJ to the co-owners of the Journal, to publish the Work in this journal and any other BMJ products and to exploit all rights, as set out in our licence.

The Submitting Author accepts and understands that any supply made under these terms is made by BMJ to the Submitting Author unless you are acting as an employee on behalf of your employer or a postgraduate student of an affiliated institution which is paying any applicable article publishing charge ("APC") for Open Access articles. Where the Submitting Author wishes to make the Work available on an Open Access basis (and intends to pay the relevant APC), the terms of reuse of such Open Access shall be governed by a Creative Commons licence – details of these licences and which Creative Commons licence will apply to this Work are set out in our licence referred to above.

Other than as permitted in any relevant BMJ Author's Self Archiving Policies, I confirm this Work has not been accepted for publication elsewhere, is not being considered for publication elsewhere and does not duplicate material already published. I confirm all authors consent to publication of this Work and authorise the granting of this licence.

For peer review only

A two-arm parallel group individually randomised Prison Pilot study of a male Remand Alcohol Intervention for Self-efficacy Enhancement: The APPRAISE Study Protocol

Aisha Holloway[1] (0000-0003-3070-3703),
Victoria Guthrie[1] (0000-0002-3920-2342),
Gillian Waller[2] (0000-0003-1873-3766),
Jamie B Smith[1,8] (0000-0003-0097-6102)
Joanne Boyd[2,3]
Sharon Mercado[4]
Pam Smith[1] (0000-0001-7660-0289),
Rosie Stenhouse[1] (0000-0002-1253-2544),
Aziz Sheikh[5] (0000-0001-7022-3056),
Richard Parker[6] (0000-0002-2658-5022),
Andrew Stoddart[6] (0000-0002-1958-3897),
Philip Conaglen[5] (0000-0002-8605-7045),
Simon Coulton[7] (0000-0002-7704-3274),
Gertraud Stadler[8] (0000-0002-1935-8200),
Kate Hunt [9] (0000-0002-5873-3632),
Jeremy Bray[10] (0000-0001-7712-160X),
Jennifer Ferguson[2] (0000-0002-1719-269X)
Arun Sondhi[11] (0000-0002-4748-5522),
Kieran Lynch[12] (0000-0002-4604-7838)
Jessica Rees[6] (0000-0002-1495-2470)
Dorothy Newbury-Birch[2] (0000-0003-0065-8649).

For peer review only

1) Nursing Studies, Doorway 6, Old Medical School, Teviot Place, University of Edinburgh, EH8 9AG, Scotland, UK.
2) School of Social Sciences, Humanities and Law, Teesside University, Middlesbrough, TS1 3BA, England, UK.
3) Humankind, Durham, DH1 3BQ, England, UK.
4) Patient and Public Involvement.
5) Usher Institute, Old Medical School, Teviot Place, University of Edinburgh, EH8 9AG, Scotland, UK.
6) Edinburgh Clinical Trials Unit, Usher Institute, Old Medical School,, Teviot Place, University of Edinburgh, EH8 9AG, Scotland, UK.
7) Centre for Health Service Research, George Allen Wing, University of Kent, Canterbury, CT2 7NZ, UK.
8) Charité - Universitätsmedizin Berlin, Health & Human Sciences, Institute of Gender in Medicine (GiM)
9) Institute for Social Marketing and Health, Faculty of Heath Sciences and Sport, University of Stirling, FK9 4LA.
10) Department of Economics, University of North Carolina at Greensboro, 462 Bryan Building, PO Box 26170, Greensboro NC 27402-6170, USA..
11) Therapeutic Solutions (Addictions) Ltd., London, UK.
12) Public Health England

Author Information

Aisha Holloway, BSc (Hons) Nursing, RGN, PhD, Professor of Nursing Studies at The University of Edinburgh.

Victoria Guthrie BSc (Hons), MSc, Senior Research Fellow at the University of Edinburgh.

Gillian Waller, BSc (Hons), MSc, PhD, Research Associate at Teesside University.

Jamie B Smith, BSc (Hons), PgDip RN, MSc, MA, Research Associate at The University of Edinburgh and Charité - Universitätsmedizin, Berlin

Joanne Boyd, Research Associate at Teesside University and Trainer for County Durham Drug and Alcohol Recovery Service.

For peer review only

Sharon Mercado BA (Hons), Former Community Justice Mentor, c/o APPRAISE study team.

Pam Smith, BNurs, RGN, MSc, PhD Professorial Fellow, Nursing Studies at The University of Edinburgh.

Rosie Stenhouse, PhD RMN Lecturer in Nursing Studies at The University of Edinburgh.

Aziz Sheikh BSc, MBBS, MSC, MD, Professor of Primary Care Research and Development and Director, Usher Institute at University of Edinburgh.

Richard Parker, Bsc (Hons), MSc, Senior Statistician at Edinburgh Clinical Trials Unit, University of Edinburgh.

Andrew Stoddart, BA (Hons), MSc, Health Economist at University of Edinburgh.

Philip Conaglen, MBChB, Prof.Dip. Marketing, MSc, MFPH, Consultant in Public Health Medicine at University of Edinburgh.

Simon Coulton BSc, MSc, Professor of Health Service Research at University of Kent.

Gertraud Stadler, PhD, Charité - Universitätsmedizin Berlin, Institute of Gender in Medicine (GiM), Prevention Research Group.

Kate Hunt MA, MSc, PhD, FRSE, Professor of Behavioural Science and Health at University of Stirling.

Jeremy Bray, BA, MA, PhD, Jefferson-Pilot Excellence, Professor, University of North Carolina at Greensboro.

Jennifer Ferguson, LLB Hons (Exempting), Graduate, Tutor PhD Researcher, Teesside University.

Arun Sondhi BA (Hons), MSc, MBA. Researcher, Therapeutic Solutions (Addictions) Ltd.

Kieran Lynch, Programme Manager Criminal Justice, Drugs, Alcohol & Tobacco, Public Health England.

Jessica Rees, PhD, Statistician at Edinburgh Clinical Trial Unit, University of Edinburgh.

Dorothy Newbury-Birch, BA (Hons), PhD, Professor of Alcohol and Public Health Research at Teesside University.

Correspondence to: Professor Aisha Holloway, BSc (Hons) Nursing, RGN, PhD

Email: aisha.holloway@ed.ac.uk

For peer review only - http://bmjopen.bmj.com/site/about/guidelines.xhtml

ABSTRACT

Introduction

The prevalence of at-risk drinking is far higher amongst those in contact with the criminal justice system (73%) than the general population (35%). However, there is little evidence on the effectiveness of alcohol brief interventions (ABIs) in reducing risky drinking amongst those in the criminal justice system, including the prison system and in particular, those on remand. Building on earlier work, APPRAISE is a pilot study designed to assess the feasibility and acceptability of an ABI, delivered to male prisoners on remand. The findings of APPRAISE should be able to provide the information required to design a future definitive randomised controlled trial (RCT).

Methods and Analysis

APPRAISE will use mixed methods, with two linked phases, across two prisons in the UK, recruiting 180 adult men on remand: 90 from Scotland and 90 from England.

Phase I will involve a 2-arm, parallel group, individually randomised pilot study. The pilot evaluation will provide data on the likely impact of APPRAISE, which will be used to inform a future definitive multi-centre RCT.

Phase II will be a process evaluation assessing how the ABI has been implemented to explore the change mechanism underpinning the ABI, and to assess the context within which the ABI is delivered.

Ethics and Dissemination

The APPRAISE protocol has been approved by the East of Scotland Research Ethics Committee (19/ES/0068), National Offender Management System (2019-240), Health Board Research and Development (2019/0268), Scottish Prison Service (SPS) research and ethics committee and by the University of Edinburgh's internal ethics department. The findings will be disseminated via peer-reviewed journal publications, presentations at local, national and international conferences, infographics and shared with relevant stakeholders through meetings and events.

Trial Registration Number ISRCTN 27417180

Keywords: alcohol, alcohol brief intervention (ABI), prison, remand prisoners.

For peer review only

Strengths and Limitations

- This study provides a unique opportunity to evaluate the feasibility of conducting trial based research with men on remand in prison, a frequently underrepresented group in research.
- A major strength is that it will assess the feasibility and acceptability of the Alcohol Brief Intervention from multiple perspectives including men on remand, prison based intervention teams, and additional stakeholder groups.
- It will provide an opportunity to identify the operational criteria necessary for a future definitive randomized control trial.
- Research in prisons is reliant on the day to day running of the establishments and this will require careful consideration and monitoring.

Introduction

Background/Rationale

The prevalence of at-risk drinking, which is defined as drinking at levels that harm a person's health, is far higher amongst those within the criminal justice system (73%) [1-5] than the general population (35%) [6]. In the United Kingdom (UK), between 51-83% of incarcerated people are classified as risky drinkers [7] and for those on remand in prison, the prevalence is between 62-68% [5]. Furthermore, alcohol dependence among those incarcerated (43%) is 10 times higher than the general population [5]. The impacts of risky drinking are significant, resulting in a significant health, economic and social burden on individuals, families and society as a whole [8]. Addressing alcohol harm in prisons, at what can be considered a 'teachable moment', could potentially reduce the risk of re-offending, reduce costs to society and help address health inequalities [9].

There is currently robust evidence from systematic reviews and meta-analyses indicating that ABIs are effective in reducing alcohol consumption amongst risky drinkers in healthcare settings (8, 9). However, there is little evidence of effectiveness of ABIs in reducing risky drinking amongst those incarcerated, particularly those on remand (10, 11), despite there being evidence in the UK that ABIs can reduce recidivism (12). In addition, ABIs have been shown to reduce recidivism within the probation setting (4).There is limited evidence around the optimum timing of delivery, recommended length, content, implementation and economic benefit of an ABI

For peer review only - http://bmjopen.bmj.com/site/about/guidelines.xhtml

in the prison setting. Furthermore, there are weaknesses within the current evidence base, with regards to the theoretical underpinnings [10, 11]. This risks an intervention being used with a weak theoretical base, poorly specified 'active' ingredients and less likely to deliver the desired outcomes. The APPRAISE pilot trial has been proposed as a way to strengthen the evidence around ABIs targeted at male remand prisoners and will seek to inform a future definitive randomised controlled trial (RCT).

Our systematic review of the efficacy of psychosocial alcohol interventions for incarcerated people [12] found that interventions within a prison have the potential to positively impact alcohol use; however, because of small numbers and different outcome measures, it was not possible to conduct a meta-analysis or generalise findings.

Notably, none of the studies focused on men on remand, with remand referring to individuals who are un-convicted, or convicted and unsentenced, held in custody awaiting trial and/or sentencing. The intervention in this proposed study, builds on previous research exploring the theoretical validity of a self-efficacy enhancing ABI [13, 14]. This intervention was originally tested within a pilot cluster-RCT, in a general hospital setting, and was able to provide evidence of a potential effect [14].

In the recently completed PRISM-A study, an ABI was developed and refined within the prison setting, working with men on remand to include a synthesis of their views, with reviews of the evidence base and theoretical underpinnings [15, 16]. PRISM-A recruited prisoners identified as risky drinkers, using the scoring >8 on the Alcohol Use Disorders Test (AUDIT) [17]. The findings showed a high prevalence of risky drinking, with 82% of participants scoring ≥8. This is comparable to other findings in the prison system in the UK [1-5], but is more than three times higher than primary care settings [6]. The study results indicated high levels of willingness of both staff and participants to engage with the intervention. In addition, support was shown for a longer intervention, which helped men on remand to develop skills and strategies that would be useful on liberation. The feedback regarding frequency and intensity of contact, identified a preference for more than one session, including additional community-based sessions, to allow participants to put their skills into practice when alcohol is more widely available. Based on learning from interviews within the PRISM-A study, the intervention for the APPRAISE pilot study includes three post-release booster sessions.

For peer review only - http://bmjopen.bmj.com/site/about/guidelines.xhtml

Following on from the positive results from the PRISM-A study, the MRC framework suggests the next step is to conduct a pilot study [18]. Therefore, a pilot study assessing the feasibility and acceptability of the ABI, in comparison to usual care, and exploring the potential effectiveness on the key parameters related to a future trial [18] will be conducted. This will provide the information required to design a definitive RCT.

Aim

The APPRAISE study aims to undertake a two-arm, parallel group, individually randomised, pilot study of a self-efficacy enhancing psychosocial alcohol intervention, for men on remand, in prison. If this pilot study is successful, it will provide the evidence to support the design of a future multi-centre RCT.

The specific study objectives are:

Objective 1: To pilot the study measures and evaluation methods to assess the feasibility of conducting a multi-centre, pragmatic, parallel group, RCT.

1a) Is it feasible to conduct a future multi-centre RCT of a self-efficacy enhancing psychosocial ABI for men on remand?

1b) Can we obtain reasonable estimates of the parameters necessary to inform the design and sample size calculation for an RCT, including standard deviations of potential continuous primary outcomes and estimates of recruitment, retention and follow-up rates?

1c) How well do participants complete the questionnaires necessary for an RCT?

1d) Can we collect economic data needed for an RCT?

1e) Can we access recidivism (as measured by 'proven' reconviction rates [19] data derived from the Police National Computer (PNC) for participants?

1f) Can we access health data from NHS data sources for participants?

Objective 2: To assess intervention fidelity

2a) What proportion of the interventions are delivered as per protocol?

2b) Is there any evidence of contamination between the two arms and/or between workers delivering the intervention?

2c) To what extent does the intervention change process variables consistent with the underpinning theory?

For peer review only - http://bmjopen.bmj.com/site/about/guidelines.xhtml

Objective 3: To qualitatively explore the feasibility and acceptability of the intervention and study measures to staff and participants on remand and on liberation

3) How acceptable are the trial and intervention procedures (including context and any barriers and facilitators) to the key stakeholders: men on remand in prison and on liberation; prison staff (including healthcare staff); commissioners; policy makers and third sector partners?

Objective 4: To assess whether operational progression criteria for conducting a future definitive RCT are met across trial arms and study sites and if so, develop a protocol for an RCT. *Operational progression criterion are based on previous research results [3]*

4a) Do the 2 prisons invited to the study agree to take part?

4b) Based on knowledge from previous data, do at least 90 eligible participants consent to take part and be randomised?

4c) Do at least 70% of participants who are allocated to intervention condition, go on to receive at least one intervention session?

4d) Do at least 60% of participants take part in the follow up assessments at 12 months across trial arms and study sites?

The APPRAISE Intervention

The ABI has four sessions, to be offered to all participants in the intervention arm. It will be delivered by existing staff at Change Grow Live (CGL) who are currently engaged in alcohol service delivery in the prison and community setting. The ABI comprises of nine elements (See Table 1 and 2) and will be delivered in 4 steps: Step 1 is 1 x 40-minute, face-to-face session, covering 9 elements, delivered by a trained CGL staff member within the prison. Steps 2, 3 and 4 are 20-minute sessions, by phone, on or as close to day 3, 7, and 21 post liberation. Exact days will be recorded to inform the process evaluation. The post liberation sessions will include elements 1 (preliminary discussion), 5 (situation-appraisal), 6 (goal setting), 7 (relapse), 8 (self-evaluation/self-reinforcement and 9 (culmination).

Self-efficacy derives from Social Cognitive Theory and has been identified as an important determinant of health behaviour and health behaviour change [20]. The four primary sources of self-efficacy information (that can be targeted through interventions) are: mastery experience; vicarious experience; verbal persuasion; and physiological state [13, 14, 20]. The ABI is designed to increase self-efficacy – through development of self-regulatory skills, self-

For peer review only - http://bmjopen.bmj.com/site/about/guidelines.xhtml

For peer review only

management and self-belief, thus enabling an individual to address their alcohol consumption. Liberation then offers participants the ability to develop and build upon these skills and self-belief through success and mastery, with their efforts leading to the adoption and maintenance of reduced alcohol consumption [13, 20]. Reducing alcohol consumption can provide a sense of achievement and success, with the overall effects of such an intervention a likely increase in the men's level of self-efficacy [13].

Control Condition (Care as Usual)

The control condition will include participants receiving care as they usually would, within the existing service provision. What constitutes care as usual will vary between participants as some participants may access no support whereas others may attend individual or group-based support sessions with CGL and/or other providers whilst in the prison or their local communities. It should be noted that participants in the intervention condition will also have access to care as usual services. Service utilization will be recorded for all participants.

Staff Training

Two members of CGL staff at each site will be trained to deliver the ABI to participants (intervention), and the remaining staff will have no training (control). This method seeks to minimize contamination and has been used in other similar studies [21, 22]. The possibility of residual contamination and the practicality of allocating staff according to randomisation, will be explored within the process evaluation, in order to inform the future RCT. Weekly de-briefs with interventionists delivering the APPRAISE ABI will also enable the exploration of possible contamination.

Intervention Delivery

The ABI has four sessions, to be offered to all participants in the intervention arm. Understanding what has been delivered and how is a key component of understanding the ABI implementation [23, 24]. As part of the process evaluation to assess implementation, measures of fidelity (quality) and dose (quantity) will be recorded and analysed. To capture fidelity, we will digitally audio record 20% of intervention sessions and code these, using the Behaviour Change Counselling Index (BECCI) [25], to assess the extent to which the essential theoretical elements of the ABI were delivered as intended. To capture dose, quantitative data from participant study records and logs

For peer review only - http://bmjopen.bmj.com/site/about/guidelines.xhtml

will be obtained to assess what proportion of interventions were offered and delivered successfully. The number of intervention sessions offered, delivered, and the length of each session will be recorded as will reasons for any unsuccessful delivery. Interviews with all implementers and 32 purposively selected participants will also be conducted at each site to identify how acceptable the trial intervention and procedures were.

Methods and Analysis

The study aligns to the MRC framework [18], using mixed methods within two linked phases conducted across two sites.

Phase I will involve a two-arm, parallel group, individually randomised pilot study (Objectives 1 and 4). The pilot evaluation will provide data on feasibility and an assessment on the likely impact of the ABI to inform the feasibility and design of a future RCT.

Phase II will comprise a process evaluation (Objectives 2 and 3), drawing on the MRC process evaluation framework to guide the planning and design of the evaluation [26]. The aim of the process evaluation is: to assess how the ABI is implemented; to undertake preliminary exploration of the change mechanism underpinning the intervention; and, to assess the delivery context of the ABI.

Baseline Assessment

Following appropriate consent procedures, baseline (TP0) data will be collected via researcher led completion of questionnaires and this will take approximately 30 minutes. Immediately after, participants will be randomised to the control or intervention arm and informed of their allocation. Participants randomised to the intervention arm will be provided with a face-to-face appointment to meet with their interventionist. This is the process used by the current providers (CGL) when engaging with individuals in the prison setting. The three telephone sessions will be delivered following release from prison by one of the trained interventionists. In addition to the outcome measures (described below), data on age, gender, marital status, educational status, and contact details will be collected.

Follow-up

For peer review only - http://bmjopen.bmj.com/site/about/guidelines.xhtml

Follow-up assessments will be conducted at 6 (TP1) and 12 months (TP2) post recruitment. These will be conducted by the researcher in person, by phone or by social media. Where participants are identified as being incarcerated at follow-up, arrangements with the relevant prison will be made to undertake the follow-up. Attempts to contact participants will be multi-faceted including telephone calls, text messages, and e-mails, based on participants' preferred method of contact, at varying times and days of the week. We will record the number of participants invited to participate; responded; were eligible; recruited; transferred; subsequently sentenced; liberated; and lost to follow-up. The number who returned to prison during the 12-month period and who completed the trial will also be recorded.

Participants and Setting

Identification of Study Participants

A suitable process of participant identification was established and found to be successful in the PRISM-A study [16, 27]. The prison induction team (consisting of prison officers and peer prisoners) will provide potential participants with a verbal account of the study and a Participant Information Sheet (PIS) during their prison induction. Researchers will liaise with relevant prison officers each week to identify potential participants. Potential participants will be contacted via prison staff; those who are willing, will meet with the researcher in an area of the prison where they can discuss issues in private, without risk of being overheard, and without risk to the interviewer or disruption to the running of the prison. The researcher will review the PIS with the participant, providing a written and verbal description of APPRAISE, answer any questions and will invite him to consider participating. The researcher will then obtain written consent from willing participants. Those who do not wish to participate, will receive no further interaction with the research staff.

Sample Size

The target population is men on remand in prison, who have been in prison for three months or less. The average time on remand is approximately nine weeks in England [28] and 4 weeks in Scotland [29]. We estimate that approximately 50% of participants will be released, while the rest will remain incarcerated; leaving 45 participants per study arm (90 in total) across the 2 sites.

For peer review only - http://bmjopen.bmj.com/site/about/guidelines.xhtml

180 adult men (18 years and over) on remand will be recruited from two sites: one within the Scottish Prison Service (SPS); Edinburgh (n=90) and the second from Her Majesty's Prison and Probation Service (HMPS); Durham, England (n=90). These sites were purposively selected following PRISM-A, as they offer geographical, socio-economic and ethnic diversity, along with differing procedures and structures [15]. Both prisons provide alcohol services via external agencies who engage with people in custody and following release. Prisons have varying induction procedures, communication mechanisms and processes, and available secure space to engage with participants. Understanding the different approaches and dynamics will be important when designing a future RCT.

This sample size was calculated to take account of the following factors [30]:

1. It will enable calculations of two-sided 95% confidence intervals (CIs) around proportions who are recruited, released, and drop-out in each study arm with half-widths of less than 0.15.

2. It exceeds the 30 per group recommendation of Lancaster et al [31] and 35 participants per group recommendation of Teare et al [32] for estimating key unknown design parameters e.g. standard deviation with sufficient precision with a continuous primary outcome.

3. It will ensure that within each arm, across each site, we satisfy the minimum 12 per group 'rule of thumb' outlined by Julious [33] for pilot trials.

Participant Eligibility Criteria

The following criteria were used in PRISM-A and were found to be appropriate.

Inclusion Criteria

Participants will be included in APPRAISE if they meet the following inclusion criteria:

- Informed consent given
- Men aged 18 years or over
- Have been in prison for three months or less on the current charge
- Score 8 or more on the AUDIT [17]
- Detained in either of the study sites within the SPS or HMPS.

For peer review only - http://bmjopen.bmj.com/site/about/guidelines.xhtml

Exclusion Criteria

Participants will be excluded from APPRAISE if they meet any of the following criteria:

- Previously recruited to APPRAISE
- Unable to give informed consent or deemed incompetent/unable to make an informed decision regarding consent
- Identified as a risk to self and/ or others by prison staff
- Judged to be under the influence of an illicit substance by prison or research staff.
- Currently taking Disulfiram (frequently referred to as Antabuse).
- On a segregative rule (under prison rules)
- Not able to understand the documents (English language) or agree to the researcher aiding their understanding.

Informed Consent

All participants will be required to provide informed consent before taking part; their decision will be entirely voluntary and based on a clear understanding of what is involved. To ensure this, all participants will receive a verbal explanation of the study, in addition to a PIS. The verbal explanation will be given by the researcher, and will cover all elements in the PIS and the consent form. Each willing participant will sign a consent form to ensure that informed consent has been obtained. Each participant will receive a copy of the form and a copy will be filed in the Investigator Site File (ISF). All participants will be given sufficient time to consider the information provided, to ask questions or to clarify anything they don't understand. Participants will be reassured that they can withdraw their consent at any time, without loss of benefits to which they otherwise would be entitled.

Confidentiality and Data Protection

All study staff will comply with the requirements of the appropriate data protection legislation (including the General Data Protection Regulation and Data Protection Act) with regard to the collection, storage, processing and disclosure of personal information. All evaluation forms, reports, and other records will maintain participant confidentiality, and will only be identifiable via a unique participant identification number. All written records will be kept in secure storage and computers used to collate the data will be password protected. Published results will not contain

For peer review only

any personal data to ensure that individuals cannot be identified. Personal data will be stored for 24 months after the study completion and following this will be securely destroyed.

Ethical Considerations

Ethical approval has been obtained from the East of Scotland NHS Research Ethics Committee, the Scottish Prisons Service Research Ethics Committee, Her Majesty's Prison and Probation Service and University of Edinburgh School of Health in Social Science Research Ethics Committee.

Due to the high-risk population group; study staff will document any occasions where a session has had to be stopped due to participant distress or where the session is terminated because the researcher of prison staff identify a risk to the researcher. This information will be communicated to the Chair within a week of it occurring. In Scotland, it will be possible to identify and document whether participants are put on a suicide risk management strategy while they are in prison. This will be monitored and any participant put onto this strategy within a week of being in contact with study staff will be flagged and reported to the Chair within 24 hours.

Blinding

Due to restrictions regarding electronic equipment and mobile phone use by researchers whilst in prison, a randomisation system, such as an Interactive Voice Response telephone system or one accessed over the web, cannot be used. Therefore, allocation will be conducted at participant level using stratified block randomisation [3, 5, 6] by site, via sealed envelopes, based on a predetermined random number allocation carried out by Edinburgh Clinical Trials Unit (ECTU) [33, 34].

Researchers will not be involved in the delivery of the active and control interventions, but will be aware of study allocation of participants as the trial progresses. Allocation concealment will be used whereby neither the person delivering the interventions, nor the participant will be aware of the study allocation until they are irrevocably entered into the trial. Both the trial statistician (RP) and health economist (JB) will be blind to group allocation and will only have details of study participants by study number.

Outcomes

For peer review only

Primary outcome measure

The proposed primary outcome measure is total alcohol consumed (in units) in a 28-day period at TP2. This is ascertained using the 28-day timeline follow back method (TLFB-28) (31-32). Two other variables can be derived from the data: percentage days abstinent, drinks per drinking day (secondary outcome measures) and total number of days where alcoholic drinks are consumed. The TLFB-28 will also be conducted by the researcher at TP 1 and takes approximately 20 minutes to complete.

Self-report secondary outcomes measures

Secondary outcome measures will be completed at TP0, TP1 and TP2. Alcohol use frequency, quantity (on a typical occasion) and binge drinking will be assessed using the AUDIT consumption questions [17]. A score of 8+ is referred to as a 'positive screen' and indicates an alcohol use disorder; hazardous drinking/increasing risk drinking (score of 8–15), harmful drinking/high risk drinking (16–19) or probable dependent drinking (20+). A score of 8 or more out of a possible 40 on the AUDIT has an established sensitivity of 92% and specificity of 94% [17] to detect those at-risk of harm from alcohol use. In order to assess the utility of using Average Drinks per Day derived from the AUDIT at 12 months as a primary outcome measure, we will randomise the order of presentation of TLFB-28 [35] and AUDIT and conduct a levels of agreement analysis, using TLFB-28 as a gold standard, to explore whether AUDIT is an acceptable proxy for TLFB-28. The order of presentation of TLFB and AUDIT will be randomised in advance by a secure remote randomisation service.

The Warwick-Edinburgh Mental Well-being scale (WEMWBS) will be used to assess mental well-being [36]. This tool uses a 5-point Likert scale, which gives a score of one to five per question, giving a minimum score of 14 and maximum score of 70. A higher WEMWBS score indicates a higher level of mental well-being [36].

Readiness to change will be measured using the Readiness to Change Ruler, which measures readiness to change drinking behaviour [7].

Self-reported alcohol self-efficacy will be measured using the Drinking Refusal Self-Efficacy Questionnaire – revised (DRSEQ-R) [37] and alcohol expectancy, using the Negative Alcohol Expectancy Questionnaire (NAEQ) [38].

For peer review only - http://bmjopen.bmj.com/site/about/guidelines.xhtml

The Euroqol (EQ-5D -5L) will be used to measure health-related quality of life and is being scoped for potential future use to generate Quality Adjusted Life Years [39]. A bespoke public sector service use questionnaire will be adapted based on the economic form 90 [40] to determine public sector costs in the domains of health and social care, criminal justice system, unemployment, and welfare.

Other secondary outcome measures

Individual level data on recidivism rates as measured by 'proven' reconviction rates [19] over a 12-month period, since TP0, derived from Police National Computer (PNC) data and use of health and social services, measured by data derived from Community Health Index (CHI)/ NHS number will be sought at TP3, along with the number of participants incarcerated at TP1 and TP2. Furthermore, data will be collected on the time spent by researchers and practitioners on APPRAISE to inform the resources needed for an RCT.

Mechanisms of impact

An intervention logic model informed by social cognitive theory has been developed. The underlying causal mechanisms will be examined, to provide increased understanding of how the intervention influences change through the quantitative assessment of key behavioural markers (mediators) of change, i.e. self-reported self-efficacy, using the Drinking Refusal Self-Efficacy Questionnaire- Revised (DRSEQ-R) [37] outcome, and alcohol expectancy, using the Negative Alcohol Expectancy Questionnaire (NAEQ) [38]. These behavioural markers of change will be recorded at TP0, TP1 and TP2 and an exploratory prognostic analysis will be undertaken to explore the nature of change within these domains and their relationship to the outcome. The relationship between alcohol consumption, receipt of the intervention and reoffending will also be assessed. In addition, we will be interviewing 32 men who took part in the study (16 at each site), eight intervention staff from CGL, and 10 additional stakeholders. The qualitative exploration of participant responses to and interactions with the intervention will be undertaken, to identify unanticipated pathways and consequences at 12 months follow up.

Data Analysis

For peer review only - http://bmjopen.bmj.com/site/about/guidelines.xhtml

The progress of participants through the APPRAISE pilot trial will be presented in accordance with the CONSORT guidelines [41] to allow descriptions of key parameters required for a future RCT; eligibility rates, consent, adherence, retention at follow-up and data completeness of outcome measures.

The statistical analyses will be primarily descriptive, providing an estimate of eligibility, recruitment, intervention delivery and retention rates within the study population. These parameters will inform the power calculations for a future RCT and confirm or identify the need for modifications in relation to other aspects of the trial design, in particular the acceptability of study processes and outcome measure to participants and staff. Data pertaining to the flow of participants through the APPRAISE pilot trial will include numbers screened, prevalence of the target condition, numbers providing contact details, numbers eligible and willing to consent and numbers followed up successfully at TP1 and TP2. In addition, data completeness for each of the instruments and any potential bias in the follow-up data will be ascertained.

Analysis of the primary outcome, (total consumption in alcohol units in the past 28-days at TP2 using the TLFB-28) will also provide estimates of the variance within and between groups for exploration of sample size calculations for an RCT. Furthermore, we will calculate the variance of all primary and secondary outcome measures within groups to explore their utility in a definitive study. If access to PNC data is possible, proven reconviction rates will be measured by the number of offences committed by each treated participant againt the number committed in the comparison group. Time-to-event analysis will also explore differences in offending patterns.

Calculation of between trial-arm differences (with 95% confidence intervals) will provide an indication of the likely effect sizes that will be observed in a future definitive trial.

For the primary outcome analysis, the mean difference (with 95% confidence interval) in alcohol units consumed between the two randomisation groups will be calculated, both overall and stratified by site. A complete cases analysis will be used, but a sensitivity analysis will also be incorporated to explore the effects of missing data. This will involve using multiple imputation for the primary outcome in a supplementary analysis. In addition, we will add and subtract one-unit differences (alcohol units) to the missing data as part of a sensitivity analysis to explore how the 95% CIs change depending on our assumptions about the missing data (bearing in mind that the imputed data cannot be less than zero). This is similar to the sensitivity analysis method

For peer review only - http://bmjopen.bmj.com/site/about/guidelines.xhtml

suggested in White et al. (2011) which involves determining how large an amount can *"be added to or subtracted from the imputed data without changing the clinical interpretation of the trial".* We will adopt a similar approach to determine how sensitive the conclusions of the APPRAISE pilot trial (and the decision about whether to proceed with an RCT) are to our assumptions about missing data.

A Statistical Analysis Plan will be written and finalised prior to analysis.

Health Economic Evaluation

Full details of all health economic analyses will be specified in a Health Economic Analysis Plan, authored by the trial health economist(s), and signed off by the PI prior to analysis.

In brief, this will scope the feasibility of conducting a future health economic evaluation alongside any future trial. A bespoke survey of public sector service use will be adapted from the Economic Form 90 [40] and tested in the patient population. We will also scope the potential for use of the EQ-5D-5L relative to other health outcome measures being used in the trial. The rates of response and completeness of questionnaires will be reported in each case and a dry run costing/QALY generation exercise will be undertaken to identify any practical issues in using these instruments. The dry run costing exercise will include a detailed scoping of the cost elements relating directly to the introduction and running of the intervention though consultation with key staff involved in the implementation. Finally the potential for longer term modelling of outcomes will be scoped through consultation with experts and non-systematic searches of the literature for similar work or anticipated parameters. These will form the basis for a short report to aid the development of a future protocol for a follow up trail.

Additional Analyses

All interview data will be transcribed and thematic analysis undertaken in order to fulfil the objectives 1d, 2c, 3a. Braun and Clarke's thematic analysis framework will guide this, as it is congruent with the theoretical perspective of the study [42]. To facilitate an understanding of each stakeholder group's perspective, the data will initially be analysed within group. The rationale for undertaking the analysis within each group is to enable the targeting of remedial actions within a future RCT. Themes running across the data, in relation to each question, will then be collated,

For peer review only

providing a multi-perspective understanding of the stakeholder experience for each element of the intervention/study.

The qualitative analysis will provide evidence to verify the feasibility and acceptability of the ABI to participants, specifically elements 5-8. It will also inform the process evaluation in Phase 2 to better understand and explain the social processes of the intervention, through the lens of implementation theory, Normalisation Process Theory [43-46].

Monitoring

An APPRAISE Trial Steering Committee (TSC) has been set up to monitor the implementation, to provide an independent assessment of the data analysis and to determine if a future trial is merited.

The TSC has the following objectives:

- Provide supervision of the trial on behalf of the sponsor and funder and ensure it is conducted to rigorous standards.
- Monitor the ongoing progress and adherence to protocol.
- Consider new information of relevance to the research question.
- Provide advice, through the chair, to the chief investigator and funder on all appropriate aspects.
- Provide evidence to support extension requests.

In addition to the TSC, the chair plus three TSC members (specialising in health economics, statistics, and qualitative methodology and analysis) have formed a subcommittee responsible for data monitoring and ethics (DMEC).

The DMEC subcommittee has the following objectives:

- To monitor the data and make a recommendation to the TSC on whether there are any ethical or safety reasons why the trial should not continue.
- To ensure that the safety, rights, and well-being of participants remains paramount.

The sub-committee will also consider the need for any interim analysis and are the only body who, if necessary, will have access to the unblinded data.

For peer review only

For peer review only - http://bmjopen.bmj.com/site/about/guidelines.xhtml

Data Management

Data from the second study site (Durham) will be anonymised and individual's data will only be identifiable by their screening number. All hard copy data will be sent to the coordinating site (University of Edinburgh) by secure courier, where it will be kept in a locked filing cabinet, with restricted access. Personal and sensitive/ wider data will be transported separately to each other in a secure manner. No personal data will be transferred outside the UK, or stored or collected on computer servers outside the UK.

Audio files will be uploaded securely to a password protected site by the researchers where the approved transcription company will be able to access the file. Transcribed files will be returned to the researchers using the same secure platform and will be downloaded and stored securely in folders on Edinburgh University's password- protected, server. Following a quality check to ensure the accuracy of the transcribed files, all audio recordings will be destroyed.

Dissemination

Our dissemination plan will include local, national and international avenues. The findings will be prepared for publication via open access, peer-reviewed journal articles. In addition, findings of the APPRAISE pilot trial and any plans for a future RCT will be presented to Health and Justice Teams at Scottish Government and Public Health England, the National Prisoner Healthcare Network (Scotland) and the WHO (Health in Prisons Programme Collaborating Centre). We will ensure participants have the opportunity to engage with research findings through infographics and will feedback to the participating sites. Other dissemination opportunities include presentations at meetings, workshops, national and international conferences, or at relevant organisations and events, such as Scottish Alcohol Research Network, Scottish Health Action on Alcohol Problems and Offender Health Research Network.

For peer review only

References

1. Coulton, S., et al., *Screening for Alcohol Use in Criminal Justice Settings: An Exploratory Study.* Alcohol and Alcoholism, 2012. **47**(4): p. 423-427.
2. Brown, N., et al., *Alcohol screening and brief intervention in a policing context: A mixed methods feasibility study.* Drug and Alcohol Review, 2010. **29**(6): p. 647-654.
3. Newbury-Birch, D., et al., *Alcohol Screening and Brief Interventions for Offenders in the Probation Setting (SIPS Trial): a Pragmatic Multicentre Cluster Randomized Controlled Trial.* Alcohol and Alcoholism, 2014. **49**(5): p. 540-548.
4. Newbury-Birch, D., et al., *Sloshed and sentenced: A prevalence study of alcohol use disorders among offenders in the North East of England.* International Journal of Prisoner Health, 2009. **5**(4): p. 201-211.
5. Newbury-Birch, D., et al., *A rapid systematic review of what we know about alcohol use disorders and brief interventions in the criminal justice system.* International Journal of Prisoner Health, 2016. **12**(1): p. 57-70.
6. Funk, M., et al., *A Multicountry Controlled Trial of Strategies to Promote Dissemination and Implementation of Brief Alcohol Intervention in Primary Health Care: Findings of a World Health Organization Collaborative Study.* Journal of Studies on Alcohol, 2005. **66**(3): p. 379-388.
7. Heather, N., D. Smailes, and P. Cassidy, *Development of a Readiness Ruler for use with alcohol brief interventions.* Drug and Alcohol Dependence, 2008. **98**(3): p. 235-240.
8. WHO, *Global status report on alcohol and health*. 2011, World Health Organisation: Geneva.
9. Revolving Doors Agency, *BALANCING ACT. Addressing health inequalities among people in contact with the criminal justice system: A briefing for Directors of Public Health*. 2013: London.
10. Heather, N., *Spreading alcohol brief interventions from health care to non-health care settings: Is it justified?* Drugs: Education, Prevention and Policy, 2016. **23**(5): p. 359-364.
11. Heather, N., *Breaking new ground in the study and practice of alcohol brief interventions.* Drug and alcohol review, 2010. **29**(6): p. 584-588.
12. Newbury-Birch, D., et al., *A Systematic Review of the Efficacy of Alcohol Interventions for Incarcerated People.* Alcohol and Alcoholism, 2018. **53**(4): p. 412-425.
13. Holloway, A.S., H.E. Watson, and G. Starr, *How do we enhance problem drinkers' self-efficacy? A nurse administered intervention.* Journal of Substance Use, 2006. **11**(6): p. 375-386.
14. Holloway, A.W., H. Watson, and A. Arthur, *An evaluation of drinking refusal Self-efficacy and minimal interventions for potential problem drinkers in a general hospital setting.* Addiction, 2007. **102**: p. 1762-70.
15. Holloway, A., et al., *Alcohol Brief Interventions for male remand prisoners: Protocol for a complex intervention framework development and feasibility study.* BMJ Open, 2017.

For peer review only - http://bmjopen.bmj.com/site/about/guidelines.xhtml

16. Holloway, A., et al., *Alcohol brief interventions for male remand prisoners: a mixed-methods feasibility and acceptability study.* The Lancet, 2019. **394**: p. S53.
17. Saunders, J.B., et al., *Development of the alcohol use disorders identification test (AUDIT): WHO collaborative project on early detection of persons with harmful alcohol consumption-II.* Addiction, 1993. **88**(6): p. 791-804.
18. Watson, M., *A framework for development and evaluation of RCTs for complex interventions to improve health.* Int J Pharm Pract, 2000. **14**(4): p. 233-4.
19. Ministry of justice, *Proven re-offending statistics: definitions and measurement*, Ministry of Justice, Editor. 2011: London.
20. Bandura, A., *Health promotion from the perspective of social cognitive theory.* Psychology and health, 1998. **13**(4): p. 623-649.
21. UKATT Research Team, *Cost effectiveness of treatment for alcohol problems: findings of the randomised UK alcohol treatment trial (UKATT).* Bmj, 2005. **331**(7516): p. 544.
22. Alderson, H., et al., *Supporting Looked After Children and Care Leavers In Decreasing Drugs, and alcohol (SOLID): protocol for a pilot feasibility randomised controlled trial of interventions to decrease risky substance use (drugs and alcohol) and improve mental health of looked after children and care leavers aged 12–20 years.* Pilot and feasibility studies, 2017. **3**(1): p. 25.
23. Carroll, C., et al., *A conceptual framework for implementation fidelity.* Implementation science, 2007. **2**(1): p. 40.
24. Montgomery, P., et al., *The Oxford Implementation Index: a new tool for incorporating implementation data into systematic reviews and meta-analyses.* Journal of clinical epidemiology, 2013. **66**(8): p. 874-882.
25. Lane, C., et al., *Measuring adaptations of motivational interviewing: the development and validation of the behavior change counseling index (BECCI).* Patient education and counseling, 2005. **56**(2): p. 166-173.
26. Moore, G.F., et al., *Process evaluation of complex interventions: Medical Research Council guidance.* bmj, 2015. **350**.
27. Holloway, A., et al., *Alcohol Brief Interventions for male remand prisoners: Protocol for a complex intervention framework development and feasibility study.* BMJ Open, 2017.
28. Prison Reform Trust, *Prison: the facts - Briefings, Bromley.* Prison Reform Trust. London, 2016.
29. Justice Committee, *An Inquiry into the use of Remand in Scotland*, J. Committee, Editor. 2018: Edinburgh.
30. Parker, R.A., *Overcoming Obstacles to Deriving Sample Size Calculations: Experiences of a Biostatistician.* 2020.
31. Lancaster, G.A., S. Dodd, and P.R. Williamson, *Design and analysis of pilot studies: recommendations for good practice.* Journal of evaluation in clinical practice, 2004. **10**(2): p. 307-312.
32. Teare, M.D., et al., *Sample size requirements to estimate key design parameters from external pilot randomised controlled trials: a simulation study.* Trials, 2014. **15**(1): p. 264.

For peer review only

33. Julious, S.A., *Sample size of 12 per group rule of thumb for a pilot study.* Pharmaceutical Statistics: The Journal of Applied Statistics in the Pharmaceutical Industry, 2005. **4**(4): p. 287-291.
34. Santarius, T., et al., *Use of drains versus no drains after burr-hole evacuation of chronic subdural haematoma: a randomised controlled trial.* The Lancet, 2009. **374**(9695): p. 1067-1073.
35. Sobell, L. and M. Sobell, *Alcohol timeline followback users' manual.* Toronto, Canada: Addiction Research Foundation, 1995.
36. Tennant, R., et al., *The Warwick-Edinburgh mental well-being scale (WEMWBS): development and UK validation.* Health and Quality of life Outcomes, 2007. **5**(1): p. 63.
37. Young, R.M. and R.G. Knight, *The Drinking Expectancy Questionnaire: A revised measure of alcohol-related beliefs.* Journal of Psychopathology and Behavioral assessment, 1989. **11**(1): p. 99-112.
38. McMahon, J. and B. Jones, *The negative alcohol expectancy questionnaire.* Journal of the Association of Nurses in Substance Abuse, 1993. **12**: p. 17.
39. The EuroQol Group, *EuroQol-a new facility for the measurement of health-related quality of life.* Health policy, 1990. **16**(3): p. 199-208.
40. Bray, J.W., et al., *Measuring economic outcomes of alcohol treatment using the Economic Form 90.* Journal of studies on alcohol and drugs, 2007. **68**(2): p. 248-255.
41. Eldridge, S.M., et al., *CONSORT 2010 statement: extension to randomised pilot and feasibility trials.* bmj, 2016. **355**: p. i5239.
42. Braun, V. and V. Clarke, *Using thematic analysis in psychology.* Qualitative research in psychology, 2006. **3**(2): p. 77-101.
43. May, C. and T. Finch, *Implementation, embedding, and integration: an outline of Normalization Process Theory.* . 2009: Sociology. p. 535-54.
44. May, C.R., et al., *Evaluating complex interventions and health technologies using normalization process theory: development of a simplified approach and web-enabled toolkit.* BMC health services research, 2011. **11**(1): p. 245.
45. May, C.R., et al., *Development of a theory of implementation and integration: Normalization Process Theory.* Implementation Science, 2009. **4**(1): p. 29.
46. Murray, E., et al., *Normalisation process theory: a framework for developing, evaluating and implementing complex interventions.* BMC medicine, 2010. **8**(1): p. 63.

Acknowledgements

Contributors

AH managed the overall design and inception of the study with DNB. AH and DNB were responsible for coordinating the Scottish and English study sites, respectively. VG and GW were the study co-ordinators (VG- Scotland and GW- England). RP is the trial statistician. All co-authors contributed to the revisions and final draft of the paper.

For peer review only

Funding:

This study is funded by the NIHR Public Health Research Programme (17/44/11).

Competing Interests

None declared

Patient Consent

Not required.

Ethics Approval

The APPRAISE protocol has been approved by the East of Scotland Research Ethics Committee (19/ES/0068), National Offender Management System (2019-240), Health Board Research and Development (2019/0268), Scottish Prison Service (SPS) research and ethics committee and The University of Edinburgh's internal ethics department.

For peer review only

Table 1: Outline of APPRAISE Intervention

Element	Elements of intervention†	Enhancing Self-efficacy	Delivery method and location**
1	Preliminary discussion	Verbal persuasion	Face to Face (P)
2	Acquiring and providing information	Verbal persuasion	Face to Face (P)
3	Self-monitoring	Verbal persuasion	Face to Face (P)
4	Increasing awareness	Physiological state	Face to Face (P)
5	Situation-appraisal and appropriate coping strategies*	Vicarious experience	Face to Face (P)<br>Mobile phone (L)
6	Goal setting*	Verbal persuasion	Face to Face (P)<br>Mobile phone (L)
7	Relapse*	Performance attainment	Face to Face (P)<br>Mobile phone (L)
8	Self-evaluation/self-reinforcement*	Performance attainment	Face to Face (P)<br>Mobile phone (L)
9	Culmination	Performance attainment	Face to Face and mobile phone (L)

†(Holloway et al, 2006) *Elements 5-8 highly rated by participants in the feasibility study (PRISM-A) and will form key focus of intervention delivery by mobile phone on liberation ** P = Prison; L = Liberation

For peer review only

Table 2: APPRAISE Intervention Elements

**Element 1: Preliminary discussion**	Opening strategies
Introduction to APPRAISE study	
Introduction to APPRAISE Intervention	
Consent, confidentiality, engagement rules, trust	
**Element 2: Acquiring and providing information**	Feedback on AUDIT score
Establish perception of impact of alcohol on health and life	
Standard units of alcohol	
Recommended drinking levels	
Alcohol-related health problems	
Legal drink/drive limit	
Tips on reducing consumption	
Where to obtain information/support (prison and liberation)	
**Element 3: Self-monitoring**	Diary card – when, where, whom, type of drink, why
**Element 4: Increasing awareness**	Balance sheet – pros and cons of drinking
Physiological sensations identified	
Alternative appraisal of somatic sensations identified	
Strategies to reduce	
**Element 5: Situation-appraisal and appropriate coping strategies**	High risk situations and antecedents of over-drinking identified
Alternative coping strategies identified	
Coping strategies verbalised by participant	

For peer review only - http://bmjopen.bmj.com/site/about/guidelines.xhtml

Praise provided	
Strategies developed further through co-production	
Strategies modelled by interventionist	
Participant verbalises strategies and visualises them	
Plan for exposure/avoidance to low risk situations and high risk situations	
General control strategies: reduction in rate of drinking, sipping, low-alcohol content and alternating between soft or low-alcohol drinks	
**Element 6: Goal setting**	Setting realistic sub-goals (short-term)
Facilitating success and increasing motivation	
**Element 7: Relapse**	What happens if you relapse
What caused the relapse?	
How do I understand relapse?	
**Element 8: Self-evaluation and self-reinforcement**	Using my alcohol diary as a means of self-evaluation and self-reinforcement
Self-congratulations and rewarding my success	
What do I attribute my success to?	
**Element 9: Culmination**	Reflections and conclusions
Plans and goals reiterated and confirmed	

For peer review only

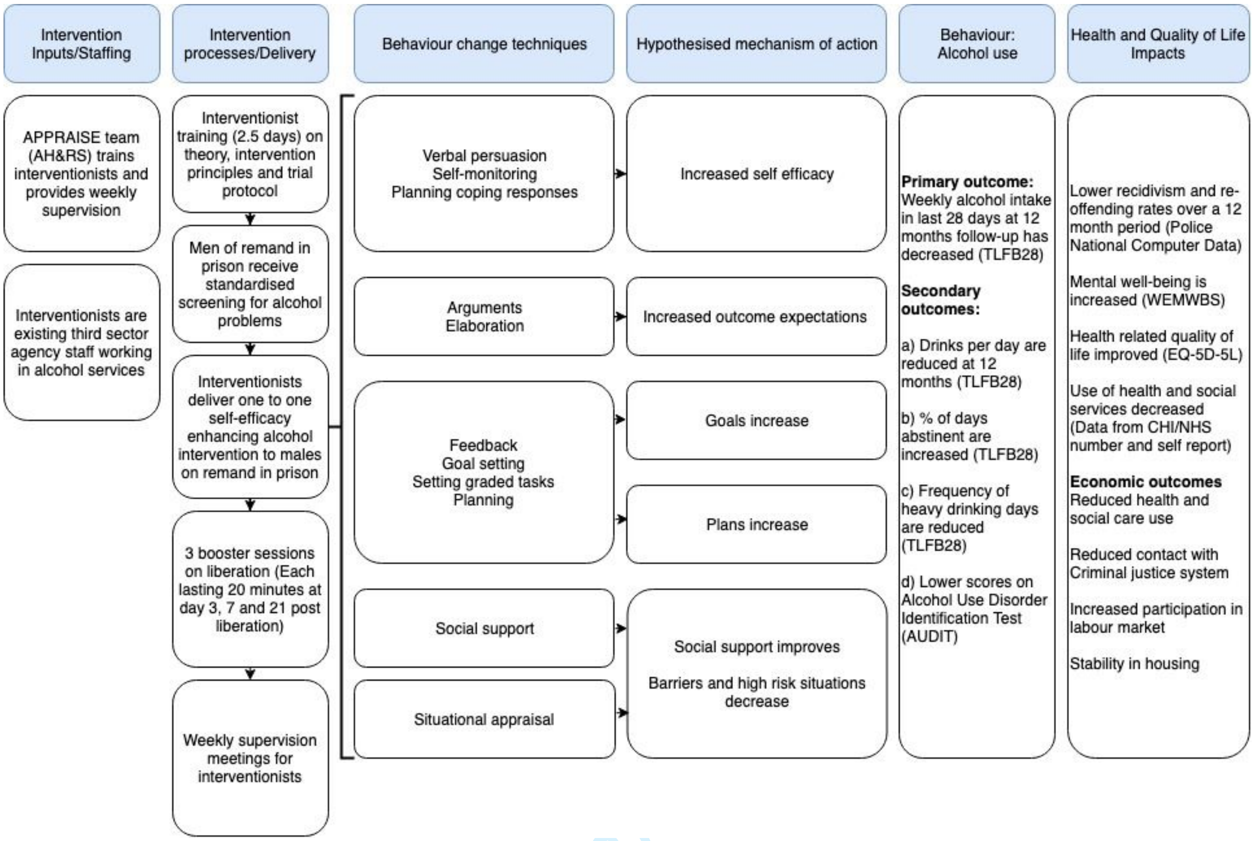

Figure 1: APPRAISE Logic Model

For peer review only - http://bmjopen.bmj.com/site/about/guidelines.xhtml

BMJ Open

A two-arm parallel group individually randomised Prison Pilot study of a male Remand Alcohol Intervention for Self-efficacy Enhancement: The APPRAISE Study Protocol

Journal:	*BMJ Open*
Manuscript ID	bmjopen-2020-040636.R1
Article Type:	Protocol
Date Submitted by the Author:	07-Dec-2020
Complete List of Authors:	Holloway, Aisha; University of Edinburgh, School of Health in Social Sciences<br>Guthrie, Victoria; University of Edinburgh, School of Health in Social Sciences<br>Waller, Gillian; Teesside University<br>Smith, Jamie; University of Edinburgh, School of Health in Social Sciences; Charite Universitatsmedizin Berlin<br>Boyd, Joanne; Teesside University; County Durham Drug and Alcohol Recovery Service, Centre for Change<br>Mercado, Sharon; University of Edinburgh, School of Health in Social Sciences<br>Smith, Pam; University of Edinburgh, School of Health in Social Science<br>Stenhouse, Rosie; University of Edinburgh, School of Health in Social Sciences<br>Sheikh, Aziz; University of Edinburgh, Division of Community Health Sciences<br>Parker, Richard; University of Edinburgh, Edinburgh Clinical Trials Unit<br>Stoddart, Andrew; The University Of Edinburgh, Edinburgh Health Services Research Unit<br>Conaglen, Philip; NHS Lothian<br>Coulton, Simon; University of Kent, Centre for Health Service Studies<br>Stadler, Gertraud; Charite Universitatsmedizin Berlin; University of Aberdeen<br>Hunt, Kate; University of Stirling, Institute of Social Marketing<br>Bray, Jeremy; University of North Carolina at Greensboro<br>Ferguson, Jennifer; Teesside University<br>Sondhi, Arun; Therapeutic Solutions,<br>Lynch, Kieran; Public Health England<br>Rees, Jessica; University of Edinburgh, Edinburgh Clinical Trials Unit<br>Newbury-Birch, Dorothy; Teesside University,
**Primary Subject Heading**:	Evidence based practice
Secondary Subject Heading:	Addiction, Health economics, Health services research, Nursing, Public health
Keywords:	PUBLIC HEALTH, Substance misuse < PSYCHIATRY, HEALTH

	ECONOMICS

SCHOLARONE™
Manuscripts

I, the Submitting Author has the right to grant and does grant on behalf of all authors of the Work (as defined in the below author licence), an exclusive licence and/or a non-exclusive licence for contributions from authors who are: i) UK Crown employees; ii) where BMJ has agreed a CC-BY licence shall apply, and/or iii) in accordance with the terms applicable for US Federal Government officers or employees acting as part of their official duties; on a worldwide, perpetual, irrevocable, royalty-free basis to BMJ Publishing Group Ltd ("BMJ") its licensees and where the relevant Journal is co-owned by BMJ to the co-owners of the Journal, to publish the Work in this journal and any other BMJ products and to exploit all rights, as set out in our licence.

The Submitting Author accepts and understands that any supply made under these terms is made by BMJ to the Submitting Author unless you are acting as an employee on behalf of your employer or a postgraduate student of an affiliated institution which is paying any applicable article publishing charge ("APC") for Open Access articles. Where the Submitting Author wishes to make the Work available on an Open Access basis (and intends to pay the relevant APC), the terms of reuse of such Open Access shall be governed by a Creative Commons licence – details of these licences and which Creative Commons licence will apply to this Work are set out in our licence referred to above.

Other than as permitted in any relevant BMJ Author's Self Archiving Policies, I confirm this Work has not been accepted for publication elsewhere, is not being considered for publication elsewhere and does not duplicate material already published. I confirm all authors consent to publication of this Work and authorise the granting of this licence.

For peer review only

A two-arm parallel group individually randomised Prison Pilot study of a male Remand Alcohol Intervention for Self-efficacy Enhancement: The APPRAISE Study Protocol

Aisha Holloway[1] (0000-0003-3070-3703),
Victoria Guthrie[1] (0000-0002-3920-2342),
Gillian Waller[2] (0000-0003-1873-3766),
Jamie B Smith[1,8] (0000-0003-0097-6102)
Joanne Boyd[2,3]
Sharon Mercado[4]
Pam Smith[1] (0000-0001-7660-0289),
Rosie Stenhouse[1] (0000-0002-1253-2544),
Aziz Sheikh[5] (0000-0001-7022-3056),
Richard Anthony Parker[6] (0000-0002-2658-5022),
Andrew Stoddart[6] (0000-0002-1958-3897),
Philip Conaglen[5] (0000-0002-8605-7045),
Simon Coulton[7] (0000-0002-7704-3274),
Gertraud Stadler[8] (0000-0002-1935-8200),
Kate Hunt [9] (0000-0002-5873-3632),
Jeremy Bray[10] (0000-0001-7712-160X),
Jennifer Ferguson[2] (0000-0002-1719-269X)
Arun Sondhi[11] (0000-0002-4748-5522),
Kieran Lynch[12] (0000-0002-4604-7838)
Jessica Rees[6] (0000-0002-1495-2470)
Dorothy Newbury-Birch[2] (0000-0003-0065-8649).

For peer review only

1) Nursing Studies, Doorway 6, Old Medical School, Teviot Place, University of Edinburgh, EH8 9AG, Scotland, UK.
2) School of Social Sciences, Humanities and Law, Teesside University, Middlesbrough, TS1 3BA, England, UK.
3) Humankind, Durham, DH1 3BQ, England, UK.
4) Patient and Public Involvement.
5) Usher Institute, Old Medical School, Teviot Place, University of Edinburgh, EH8 9AG, Scotland, UK.
6) Edinburgh Clinical Trials Unit, Usher Institute, Old Medical School,, Teviot Place, University of Edinburgh, EH8 9AG, Scotland, UK.
7) Centre for Health Service Studies, George Allen Wing, University of Kent, Canterbury, CT2 7NZ, UK.
8) Charité - Universitätsmedizin Berlin, Health & Human Sciences, Institute of Gender in Medicine (GiM)
9) Institute for Social Marketing and Health, Faculty of Heath Sciences and Sport, University of Stirling, FK9 4LA.
10) Department of Economics, University of North Carolina at Greensboro, 462 Bryan Building, PO Box 26170, Greensboro NC 27402-6170, USA..
11) Therapeutic Solutions (Addictions) Ltd., London, UK.
12) Public Health England

For peer review only

Author Information

Aisha Holloway, BSc (Hons) Nursing, RGN, PhD, Professor of Nursing Studies at The University of Edinburgh.

Dorothy Newbury-Birch, BA (Hons), PhD, Professor of Alcohol and Public Health Research at Teesside University.

Victoria Guthrie, BSc (Hons), MSc, PhD, Senior Research Fellow at the University of Edinburgh.

Gillian Waller, BSc (Hons), MSc, PhD, Research Associate at Teesside University.

Jamie B Smith, BSc (Hons), PgDip RN, MSc, MA, Research Associate at The University of Edinburgh and Charité - Universitätsmedizin, Berlin

Joanne Boyd, Research Associate at Teesside University and Trainer for County Durham Drug and Alcohol Recovery Service.

Pam Smith, BNurs, RGN, MSc, PhD Professorial Fellow, Nursing Studies at The University of Edinburgh.

Rosie Stenhouse, PhD RMN Lecturer in Nursing Studies at The University of Edinburgh.

Aziz Sheikh, BSc, MBBS, MSC, MD, Professor of Primary Care Research and Development and Director, Usher Institute at University of Edinburgh.

Richard Parker, Bsc (Hons), MSc, Senior Statistician at Edinburgh Clinical Trials Unit, University of Edinburgh.

Andrew Stoddart, BA (Hons), MSc, Health Economist at University of Edinburgh.

Philip Conaglen, MBChB, Prof.Dip. Marketing, MSc, MFPH, Consultant in Public Health Medicine at University of Edinburgh.

For peer review only

Simon Coulton, BSc, MSc, Professor of Health Services Research at University of Kent.

Gertraud Stadler, PhD, Charité - Universitätsmedizin Berlin, Institute of Gender in Medicine (GiM), Prevention Research Group.

Kate Hunt, MA, MSc, PhD, FRSE, Professor of Behavioural Science and Health at University of Stirling.

Sharon Mercado, BA (Hons), Former Community Justice Mentor, c/o APPRAISE study team.

Jeremy Bray, BA, MA, PhD, Jefferson-Pilot Excellence, Professor, University of North Carolina at Greensboro.

Jennifer Ferguson, LLB Hons (Exempting), Graduate Tutor, PhD Researcher, Teesside University.

Arun Sondhi, BA (Hons), MSc, MBA. Researcher, Therapeutic Solutions (Addictions) Ltd.

Kieran Lynch, Programme Manager Criminal Justice, Drugs, Alcohol & Tobacco, Public Health England.

Jessica Rees, PhD, Statistician at Edinburgh Clinical Trial Unit, University of Edinburgh.

Correspondence to: Professor Aisha Holloway, BSc (Hons) Nursing, RGN, PhD

Email: aisha.holloway@ed.ac.uk

For peer review only

ABSTRACT

Introduction

The prevalence of at-risk drinking is far higher amongst those in contact with the criminal justice system (73%) than the general population (35%). However, there is little evidence on the effectiveness of alcohol brief interventions (ABIs) in reducing risky drinking amongst those in the criminal justice system, including the prison system and in particular, those on remand. Building on earlier work, APPRAISE is a pilot study designed to assess the feasibility and acceptability of an ABI, delivered to male prisoners on remand. The findings of APPRAISE should be able to provide the information required to design a future definitive randomised controlled trial (RCT).

Methods and Analysis

APPRAISE will use mixed methods, with two linked phases, across two prisons in the UK, recruiting 180 adult men on remand: 90 from Scotland and 90 from England. Phase I will involve a 2-arm, parallel group, individually randomised pilot study. The pilot evaluation will provide data on the likely impact of APPRAISE, which will be used to inform a future definitive multi-centre RCT. Phase II will be a process evaluation assessing how the ABI has been implemented to explore the change mechanism underpinning the ABI, and to assess the context within which the ABI is delivered.

Ethics and Dissemination

The APPRAISE protocol has been approved by the East of Scotland Research Ethics Committee (19/ES/0068), National Offender Management System (2019-240), Health Board Research and Development (2019/0268), Scottish Prison Service (SPS) research and ethics committee and by the University of Edinburgh's internal ethics department. The findings will be disseminated via peer-reviewed journal publications, presentations at local, national and international conferences, infographics and shared with relevant stakeholders through meetings and events.

Trial Registration Number ISRCTN 27417180

Keywords: alcohol, alcohol brief intervention (ABI), prison, remand prisoners.

Strengths and Limitations

For peer review only

- This study provides a unique opportunity to evaluate the feasibility of conducting trial based research with men on remand in prison, a frequently underrepresented group in research.
- A major strength is that it will assess the feasibility and acceptability of the Alcohol Brief Intervention from multiple perspectives including men on remand, prison based intervention teams, and additional stakeholder groups.
- It will provide an opportunity to identify the operational criteria necessary for a future definitive randomized control trial.
- The unique context provides challenges for research and research activities will be influenced by the day to day running of the prisons.

For peer review only

Introduction

Background/Rationale

The prevalence of at-risk drinking, which is defined as drinking at levels that harm a person's health, is far higher amongst those within the criminal justice system (73%) [1-5] than the general population (35%) [6]. In the United Kingdom (UK), between 51-83% of incarcerated people are classified as risky drinkers [7] and for those on remand in prison, the prevalence is between 62-68% [5]. Furthermore, alcohol dependence among those incarcerated (43%) is 10 times higher than the general population [5]. The impacts of risky drinking are significant, resulting in a significant health, economic and social burden on individuals, families and society as a whole [8]. Addressing alcohol harm in prisons, at what can be considered a 'teachable moment', could potentially reduce the risk of re-offending, reduce costs to society and help address health inequalities [9].

There is currently robust evidence from systematic reviews and meta-analyses indicating that Alcohol Brief Interventions (ABIs) are effective in reducing alcohol consumption amongst at-risk drinkers in healthcare settings [8, 9]. However, there is little evidence of effectiveness of ABIs in reducing risky drinking amongst those incarcerated, particularly those on remand [10, 11],despite there being evidence in the UK that ABIs can reduce recidivism [12].

In addition, ABIs have been shown to reduce recidivism within the probation setting [13]. Our systematic review of the efficacy of psychosocial alcohol interventions for incarcerated people [10] found that interventions within a prison have the potential to positively impact alcohol use; however, because of small numbers and different outcome measures, it was not possible to conduct a meta-analysis or generalise findings. Notably, none of the studies focused on men on remand, with remand referring to individuals who are un-convicted, or convicted and unsentenced, held in custody awaiting trial and/or sentencing. The intervention in this proposed study, builds on previous research exploring the theoretical validity of a self-efficacy enhancing ABI [14, 15]. This intervention was originally tested within a pilot cluster-RCT, in a general hospital setting, and was able to provide evidence of a potential effect [15].

In the recently completed PRISM-A study, an ABI was developed and refined within the prison setting, working with men on remand to include a synthesis of their views, with reviews of

For peer review only - http://bmjopen.bmj.com/site/about/guidelines.xhtml

the evidence base and theoretical underpinnings [16, 17]. The findings also showed a high prevalence of at-risk drinking, with 82% of participants scoring ≥8 on the Alcohol Use Disorders Test (AUDIT) [17]. This is comparable to other findings in the prison system in the UK [1-5], but is more than three times higher than primary care settings [6]. The study results indicated high levels of willingness of both staff and participants to engage with the intervention. In addition, support was shown for a longer intervention, which helped men on remand to develop skills and strategies that would be useful on liberation. The feedback regarding frequency and intensity of contact, identified a preference for more than one session, including additional community-based sessions, to allow participants to put their skills into practice when alcohol is more widely available. Based on learning from interviews within the PRISM-A study, the intervention for the APPRAISE pilot study includes three post-release booster sessions.

However, there is limited evidence around the optimum timing of delivery, recommended length, content, implementation and economic benefit of an ABI in the prison setting. Furthermore, there are weaknesses within the current evidence base, with interventions not having theoretical underpinnings [11, 18]. This risks an intervention being used with a weak theoretical base, poorly specified 'active' ingredients and less likely to deliver the desired outcomes.

Following on from the positive results from the PRISM-A study, the MRC framework suggests the next step is to conduct a pilot study [19]. The APPRAISE pilot trial has therefore been proposed as a way to strengthen the evidence around ABIs targeted at male remand prisoners. It will seek to assess the feasibility and acceptability of the ABI, in comparison to usual care, it will explore the potential effectiveness of the key parameters and will aim to provide the information required to be able to design a definitive RCT.

Aim

The APPRAISE study aims to undertake a two-arm, parallel group, individually randomised, pilot study of a self-efficacy enhancing psychosocial alcohol intervention, for men on remand, in prison. If this pilot study is successful, it will provide the evidence to support the design of a future multi-centre RCT.

The specific study objectives are:

For peer review only - http://bmjopen.bmj.com/site/about/guidelines.xhtml

Objective 1: To pilot the study measures and evaluation methods to assess the feasibility of conducting a multi-centre, pragmatic, parallel group, RCT.

1a) Is it feasible to conduct a future multi-centre RCT of a self-efficacy enhancing psychosocial ABI for men on remand?

1b) Can we obtain reasonable estimates of the parameters necessary to inform the design and sample size calculation for an RCT, including standard deviations of potential continuous primary outcomes and estimates of recruitment, retention and follow-up rates?

1c) Can we determine, based on rates of missing data, whether the questionnaires are appropriate for the target population?

1d) Can we collect economic data needed for an RCT?

1e) Can we access recidivism (as measured by 'proven' reconviction rates [20] data derived from the Police National Computer (PNC) for participants)?

1f) Can we access health data from NHS data sources for participants?

Objective 2: To assess intervention fidelity

2a) What proportion of the interventions are delivered as per protocol?

2b) Is there any evidence of contamination between the two arms and/or between workers delivering the intervention?

2c) To what extent does the intervention change process variables consistent with the underpinning theory?

Objective 3: To qualitatively explore the feasibility and acceptability of the intervention and study measures to staff and participants on remand and on liberation

3) How acceptable are the trial and intervention procedures (including context and any barriers and facilitators) to the key stakeholders: men on remand in prison and on liberation; prison staff (including healthcare staff); commissioners; policy makers and third sector partners?

Objective 4: To assess whether operational progression criteria for conducting a future definitive RCT are met across trial arms and study sites and if so, develop a protocol for an RCT. *Operational progression criterion are based on previous research results [3]*

4a) Do the 2 prisons invited to the study agree to take part?

4b) Based on knowledge from previous data, do at least 90 eligible participants consent to take part and be randomised?

For peer review only - http://bmjopen.bmj.com/site/about/guidelines.xhtml

4c) Do at least 70% of participants who are allocated to intervention condition, go on to receive at least one intervention session?

4d) Do at least 60% of participants take part in the follow up assessments at 12 months across trial arms and study sites?

Methods and Analysis

Study Design

The study aligns to the MRC framework [19], using mixed methods within two linked phases conducted across two sites.

Phase I will involve a two-arm, parallel group, individually randomised pilot study (Objectives 1 and 4). The pilot evaluation will provide data on feasibility and an assessment on the likely impact of the ABI to inform the feasibility and design of a future RCT.

Phase II will comprise a process evaluation (Objectives 2 and 3), drawing on the MRC process evaluation framework to guide the planning and design of the evaluation [21]. The aim of the process evaluation is: to assess how the ABI is implemented; to undertake preliminary exploration of the change mechanism underpinning the intervention; and, to assess the delivery context of the ABI.

Participants and Setting

Identification of Study Participants

A suitable process of participant identification was established and found to be successful in the PRISM-A study [17, 22]. The prison induction team (consisting of prison officers and peer prisoners) will provide potential participants with a verbal account of the study and a Participant Information Sheet (PIS) during their prison induction. Researchers will liaise with relevant prison officers each week to identify potential participants. Potential participants will be contacted via prison staff; those who are willing, will meet with the researcher in an area of the prison where they can discuss issues in private, without risk of being overheard, and without risk to the interviewer or disruption to the running of the prison. The researcher will review the PIS with the participant, providing a written and verbal description of APPRAISE, answer any

For peer review only

questions and will invite him to consider participating. The researcher will then obtain written consent from willing participants. Those who do not wish to participate, will receive no further interaction with the research staff.

Sample Size

180 adult men (18 years and over) on remand will be recruited from two sites: one within the Scottish Prison Service (SPS); Edinburgh (n=90) and the second from Her Majesty's Prison and Probation Service (HMPS); Durham, England (n=90). These sites were purposively selected following PRISM-A, as they offer geographical, socio-economic and ethnic diversity, along with differing procedures and structures [16]. Both prisons provide alcohol services via external agencies who engage with people in custody and following release. Prisons have varying induction procedures, communication mechanisms and processes, and available secure space to engage with participants. Understanding the different approaches and dynamics will be important when designing a future RCT.

The target population is men on remand in prison, who have been in prison for three months or less. The average time on remand is approximately nine weeks in England [23] and 4 weeks in Scotland [24]. We estimate that approximately 50% of participants will be released, while the rest will remain incarcerated; leaving 45 participants per study arm (90 in total) across the 2 sites.

This sample size was calculated to take account of the following factors [25]:

1. It will enable calculations of two-sided 95% confidence intervals (CIs) around proportions who are recruited, released, and drop-out in each study arm with half-widths of less than 0.15.

2. It exceeds the 30 per group recommendation of Lancaster et al [26] and 35 participants per group recommendation of Teare et al [27] for estimating key unknown design parameters e.g. standard deviation with sufficient precision with a continuous primary outcome.

3. It will ensure that within each arm, across each site, we satisfy the minimum 12 per group 'rule of thumb' outlined by Julious [28] for pilot trials.

Participant Eligibility Criteria

For peer review only - http://bmjopen.bmj.com/site/about/guidelines.xhtml

The following criteria were used in PRISM-A and were found to be appropriate.

Inclusion Criteria

Participants will be included in APPRAISE if they meet the following inclusion criteria:

- Informed consent given
- Men aged 18 years or over
- Have been in prison for three months or less on the current charge
- Score 8 or more on the AUDIT [29]
- Detained in either of the study sites within the SPS or HMPS.

Exclusion Criteria

Participants will be excluded from APPRAISE if they meet any of the following criteria:

- Previously recruited to APPRAISE
- Unable to give informed consent or deemed incompetent/unable to make an informed decision regarding consent
- Identified as a risk to self and/ or others by prison staff
- Judged to be under the influence of an illicit substance by prison or research staff.
- Currently taking Disulfiram (frequently referred to as Antabuse).
- On a segregative rule (under prison rules)
- Not able to understand the documents (English language) or agree to the researcher aiding their understanding.

Informed Consent

All participants will be required to provide informed consent before taking part; their decision will be entirely voluntary and based on a clear understanding of what is involved. To ensure this, all participants will receive a verbal explanation of the study, in addition to a PIS. The verbal explanation will be given by the researcher, and will cover all elements in the PIS and the consent form. Each willing participant will sign a consent form to ensure that informed consent has been obtained. Each participant will receive a copy of the form and a copy will be filed in the Investigator Site File (ISF). All participants will be given sufficient time to consider the information provided, to ask questions and to clarify anything they don't understand. Participants will be

For peer review only - http://bmjopen.bmj.com/site/about/guidelines.xhtml

reassured that they can withdraw their consent at any time, without loss of benefits to which they otherwise would be entitled.

The APPRAISE Intervention

The ABI has four sessions, to be offered to all participants in the intervention arm. It will be delivered by existing staff at Change Grow Live (CGL) who are currently engaged in alcohol service delivery in the prison and community setting. The ABI comprises of nine elements (See Table 1 and 2) and will be delivered in 4 steps: Step 1 is 1 x 40-minute, face-to-face session, covering 9 elements, delivered by a trained CGL staff member within the prison. Steps 2, 3 and 4 are 20-minute sessions, by phone, on or as close to day 3, 7, and 21 post liberation. Exact days will be recorded to inform the process evaluation. The post liberation sessions will include elements 1 (preliminary discussion), 5 (situation-appraisal), 6 (goal setting), 7 (relapse), 8 (self-evaluation/self-reinforcement and 9 (culmination).

Self-efficacy derives from Social Cognitive Theory and has been identified as an important determinant of health behaviour and health behaviour change [30]. The four primary sources of self-efficacy information (that can be targeted through interventions) are: mastery experience; vicarious experience; verbal persuasion; and physiological state [14, 15, 30]. The ABI is designed to increase self-efficacy – through development of self-regulatory skills, self-management and self-belief, thus enabling an individual to address their alcohol consumption. Liberation then offers participants the ability to develop and build upon these skills and self-belief through success and mastery, with their efforts leading to the adoption and maintenance of reduced alcohol consumption [14, 30]. Reducing alcohol consumption can provide a sense of achievement and success, with the overall effects of such an intervention a likely increase in the men's level of self-efficacy [14].

Staff Training

Two members of CGL staff at each site will be trained to deliver the ABI to participants (intervention), and the remaining staff will have no training (control). This method seeks to minimize contamination and has been used in other similar studies [31, 32]. The possibility of residual contamination and the practicality of allocating staff according to randomisation, will be explored within the process evaluation, in order to inform the future

For peer review only - http://bmjopen.bmj.com/site/about/guidelines.xhtml

RCT. Weekly de-briefs with interventionists delivering the APPRAISE ABI will also enable the exploration of possible contamination.

Intervention Delivery

The ABI has four sessions, to be offered to all participants in the intervention arm. Understanding what has been delivered and how is a key component of understanding the ABI implementation [33, 34]. As part of the process evaluation to assess implementation, measures of fidelity (quality) and dose (quantity) will be recorded and analysed. To capture fidelity, we will digitally audio record 20% of intervention sessions and code these, using the Behaviour Change Counselling Index (BECCI) [35], to assess the extent to which the essential theoretical elements of the ABI were delivered as intended. To capture dose, quantitative data from participant study records and logs will be obtained to assess what proportion of interventions were offered and delivered successfully. The number of intervention sessions offered, delivered, and the length of each session will be recorded as will reasons for any unsuccessful delivery. Interviews with all implementers and 32 purposively selected participants will also be conducted at each site to identify how acceptable the trial intervention and procedures were.

Control Condition (Care as Usual)

The control condition will include participants receiving care as they usually would, within the existing service provision. What constitutes care as usual will vary between participants as some participants may access no support whereas others may attend individual or group-based support sessions with CGL and/or other providers whilst in the prison or their local communities. It should be noted that participants in the intervention condition will also have access to care as usual services. Service utilization will be recorded for all participants.

Data Collection

Baseline Assessment

Following appropriate consent procedures, baseline (TP0) data will be collected via researcher led completion of questionnaires and this will take approximately 30 minutes. Immediately after, participants will be randomised to the control or intervention arm and informed of their allocation. Participants randomised to the intervention arm will be provided with a face-to-face appointment

For peer review only

to meet with their interventionist. This is the process used by the current providers (CGL) when engaging with individuals in the prison setting. The three telephone sessions will be delivered following release from prison by one of the trained interventionists. In addition to the outcome measures (described below), data on age, gender, marital status, educational status, and contact details will be collected.

Blinding

Due to restrictions regarding electronic equipment and mobile phone use by researchers whilst in prison, a randomisation system, such as an Interactive Voice Response telephone system or one accessed over the web, cannot be used. Allocation will be conducted at participant level using random permuted blocks of variable size [3, 5, 6] stratified by site using sealed envelopes, based on a predetermined random number allocation carried out by Edinburgh Clinical Trials Unit (ECTU) [28, 36].

Researchers will not be involved in the delivery of the active and control interventions, but will be aware of study allocation of participants as the trial progresses. Allocation concealment will be used whereby neither the person delivering the interventions, nor the participant will be aware of the study allocation until they are irrevocably entered into the trial. Both the trial statistician (RP) and health economist (JB) will be blind to group allocation and will only have details of study participants by study number.

Follow-up

Follow-up assessments will be conducted at 6 (TP1) and 12 months (TP2) post recruitment. The original aim was for these to be conducted by the researcher either in person or by telephone. In light of COVID-19 restrictions, follow-up assessments have been adapted to also enable self-completion using hard copies or electronic versions. Where participants are identified as being incarcerated at follow-up, arrangements with the relevant prison will be made to undertake the follow-up. Attempts to contact participants will be multi-faceted including telephone calls, text messages, and e-mails, based on participants' preferred method of contact, at varying times and days of the week. We will record the number of participants invited to participate; responded; were eligible; recruited; transferred; subsequently sentenced; liberated; and lost to follow-up. The number who returned to prison during the 12-month period and who completed the trial will also be recorded.

For peer review only - http://bmjopen.bmj.com/site/about/guidelines.xhtml

Outcomes

Primary outcome measure

The proposed primary outcome measure is alcohol consumed, in units where one unit equals 8g or 10ml of ethanol, per week will be derived from the frequency and quantity of alcohol consumption questions in the extended version of AUDIT using a formula developed and employed in a number of randomised controlled trials of interventions for alcohol consumption [37, 38]. A score of 8+ is referred to as a ‘positive screen’ and indicates drinking at hazardous (score of 8–15), harmful (score of 16-19), or probable dependent level (score of 20+).A score of 8 or more out of a possible 40 on the AUDIT has an established sensitivity of 92% and specificity of 94% [29] to detect those at-risk of harm from alcohol use. The AUDIT will be administered at TP0, TP1 and TP2.

Self-report secondary outcomes measures

Secondary outcome measures will be completed at TP0, TP1 and TP2.

The Warwick-Edinburgh Mental Well-being scale (WEMWBS) will be used to assess mental well-being [39]. This tool uses a 5-point Likert scale, which gives a score of one to five per question, giving a minimum score of 14 and maximum score of 70. A higher WEMWBS score indicates a higher level of mental well-being [39].

Readiness to change will be measured using the Readiness to Change Ruler, which measures readiness to change drinking behaviour [7].

Self-reported alcohol self-efficacy will be measured using the Drinking Refusal Self-Efficacy Questionnaire – revised (DRSEQ-R) [40] and alcohol expectancy, using the Negative Alcohol Expectancy Questionnaire (NAEQ) [41].

The Euroqol (EQ-5D -5L) will be used to measure health-related quality of life and is being scoped for potential future use to generate Quality Adjusted Life Years [42]. A bespoke public sector service use questionnaire will be adapted based on the economic form 90 [43] to determine

For peer review only

public sector costs in the domains of health and social care, criminal justice system, unemployment, and welfare.

Other secondary outcome measures

Attempts will be made to access data from the Police National Computer (PNC) in order to collect individual level data on recidivism rates as measured by 'proven' reconviction rates [20] over a 12-month period, since TP0. The number of participants incarcerated at TP1 and TP2 will also be recorded.

Individual level data relating to health and social care use will also be sought. If access is possible, data derived from Community Health Index (CHI)/ NHS number at TP3 will be used to validate the bespoke public sector service use questionnaire. Furthermore, data will be collected on the time spent by researchers and practitioners on APPRAISE to inform the resources needed for a definitive RCT.

Mechanisms of impact

An intervention logic model (Figure 1) informed by social cognitive theory has been developed. The underlying causal mechanisms will be examined, to provide increased understanding of how the intervention influences change through the quantitative assessment of key behavioural markers (mediators) of change, i.e. self-reported self-efficacy, using the Drinking Refusal Self-Efficacy Questionnaire- Revised (DRSEQ-R) [40] and alcohol expectancy, using the Negative Alcohol Expectancy Questionnaire (NAEQ) [41]. These behavioural markers of change will be recorded at TP0, TP1 and TP2 and an exploratory prognostic analysis will be undertaken to explore the nature of change within these domains and their relationship to the primary outcome. The relationship between alcohol consumption, receipt of the intervention and reoffending will also be assessed. In addition, we will be interviewing 32 men who took part in the study (16 at each site), eight intervention staff from CGL, and 10 additional stakeholders. The qualitative exploration of participant responses to and interactions with the intervention will be undertaken, to identify unanticipated pathways and consequences at 12 months follow up.

Data Analysis

For peer review only

The progress of participants through the APPRAISE pilot trial will be presented in accordance with the CONSORT guidelines [44] to allow descriptions of key parameters required for a future RCT; eligibility rates, consent, adherence, retention at follow-up and data completeness of outcome measures.

The statistical analyses will be primarily descriptive, providing an estimate of the proportion of those eligible, consenting, adhering to the intervention and retention rates at 6 and 12 months. These parameters will inform the power calculations for a future RCT and confirm or identify the need for modifications in relation to other aspects of the trial design, in particular the acceptability of study processes and outcome measures to participants and staff. Data pertaining to the flow of participants through the APPRAISE pilot trial will include numbers screened, prevalence of the target condition, numbers providing contact details, numbers eligible and willing to consent and numbers followed up successfully at TP1 and TP2. In addition, data completeness for each of the instruments and any potential bias in the follow-up data will be ascertained.

Analysis of the primary outcome, will also provide estimates of the variance within and between groups for exploration of sample size calculations for a definitive RCT. Furthermore, we will calculate the variance of all primary and secondary outcome measures within groups to explore their utility in a definitive study. If access to PNC data is possible, proven reconviction rates will be measured by the number of offences committed by each treated participant against the number committed in the comparison group. Time-to-event analysis will also explore differences in offending patterns.

Calculation of between trial-arm differences (with 95% confidence intervals) will provide an indication of the likely effect sizes that will be observed in a future definitive trial.

For the primary outcome analysis, the mean difference (with 95% confidence interval) in alcohol units consumed between the two randomisation groups will be calculated, both overall and stratified by site. A complete cases analysis will be used, but a sensitivity analysis will also be incorporated to explore the effects of missing data. This will involve using multiple imputation for the primary outcome in a supplementary analysis, provided that the valid sample size is sufficient to support this. In addition, we will add and subtract one-unit differences (alcohol units) to the missing data as part of a sensitivity analysis to explore how the 95% CIs change depending on our assumptions about the missing data (bearing in mind that the imputed data cannot be less

For peer review only

than zero). This is similar to the sensitivity analysis method suggested in White et al. (2011) which involves determining how large an amount can *"be added to or subtracted from the imputed data without changing the clinical interpretation of the trial".* We will adopt a similar approach to determine how sensitive the conclusions of the APPRAISE pilot trial (and the decision about whether to proceed with an RCT) are to our assumptions about missing data.

A Statistical Analysis Plan will be written and finalised prior to analysis.

Health Economic Evaluation

Full details of all health economic analyses will be specified in a Health Economic Analysis Plan, authored by the trial health economist(s), and signed off by the PI prior to analysis.

In brief, this will scope the feasibility of conducting a future health economic evaluation alongside any future trial. A bespoke survey of public sector service use will be adapted from the Economic Form 90 [43] and tested in the patient population. We will also scope the potential for use of the EQ-5D-5L relative to other health outcome measures being used in the trial. The rates of response and completeness of questionnaires will be reported in each case and a dry run costing/QALY generation exercise will be undertaken to identify any practical issues in using these instruments. The dry run costing exercise will include a detailed scoping of the cost elements relating directly to the introduction and running of the intervention though consultation with key staff involved in the implementation. Finally the potential for longer term modelling of outcomes will be scoped through consultation with experts and non-systematic searches of the literature for similar work or anticipated parameters. These will form the basis for a short report to aid the development of a future protocol for a follow up trail.

Additional Analyses

All interview data will be transcribed and thematic analysis undertaken in order to fulfil the objectives 1d, 2c, 3a. Braun and Clarke's thematic analysis framework will guide this, as it is congruent with the theoretical perspective of the study [45]. To facilitate an understanding of each stakeholder group's perspective, the data will initially be analysed within group. The rationale for undertaking the analysis within each group is to enable the targeting of remedial actions within a future RCT. Themes running across the data, in relation to each question, will then be collated,

For peer review only - http://bmjopen.bmj.com/site/about/guidelines.xhtml

providing a multi-perspective understanding of the stakeholder experience for each element of the intervention/study.

The qualitative analysis will provide evidence to verify the feasibility and acceptability of the ABI to participants, specifically elements 5-8. It will also inform the process evaluation in Phase 2 to better understand and explain the social processes of the intervention, through the lens of implementation theory, Normalisation Process Theory [46-49].

Monitoring

An APPRAISE Trial Steering Committee (TSC) has been set up to monitor the implementation, to provide an independent assessment of the data analysis and to determine if a future trial is merited.

The TSC has the following objectives:

- Provide supervision of the trial on behalf of the sponsor and funder and ensure it is conducted to rigorous standards.
- Monitor the ongoing progress and adherence to protocol.
- Consider new information of relevance to the research question.
- Provide advice, through the chair, to the chief investigator and funder on all appropriate aspects.
- Provide evidence to support extension requests.

In addition to the TSC, the chair plus three TSC members (specialising in health economics, statistics, and qualitative methodology and analysis) have formed a subcommittee responsible for data monitoring and ethics (DMEC).

The DMEC subcommittee has the following objectives:

- To monitor the data and make a recommendation to the TSC on whether there are any ethical or safety reasons why the trial should not continue.
- To ensure that the safety, rights, and well-being of participants remains paramount.

The sub-committee will also consider the need for any interim analysis and are the only body who, if necessary, will have access to the unblinded data.

For peer review only

Data Management

Data from both study sites will be anonymised and individual's data will only be identifiable by their unique screening number. All of the hard copy data from the Durham site will be sent to the coordinating site, The University of Edinburgh, by secure courier, where all of the data will then be stored in a locked filing cabinet, with restricted access. Personal or sensitive data will be transported separately between sites in a secure manner, using password protected files. No personal data will be transferred outside the UK, or stored or collected on computer servers outside the UK.

Audio files will be uploaded securely to a password protected site by the researchers, where the approved transcription company will be able to access the files. Transcribed files will be returned to the researchers using the same secure platform and will be downloaded and stored securely in folders on the University of Edinburgh's password- protected, server. Following a quality check to ensure the accuracy of the transcribed files, all audio recordings will be securely destroyed.

Confidentiality and Data Protection

All study staff will comply with the requirements of the appropriate data protection legislation (including the General Data Protection Regulation and Data Protection Act) with regard to the collection, storage, processing and disclosure of personal information. All evaluation forms, reports, and other records will maintain participant confidentiality, and will only be identifiable via a unique participant identification number. All written records will be kept in secure storage and computers used to collate the data will be password protected. Published results will not contain any personal data to ensure that individuals cannot be identified. Personal data will be stored for 24 months after the study completion and following this will be securely destroyed.

Ethical Considerations

Ethical approval has been obtained from the East of Scotland NHS Research Ethics Committee, the Scottish Prisons Service Research Ethics Committee, Her Majesty's Prison and Probation Service and University of Edinburgh School of Health in Social Science Research Ethics Committee.

For peer review only

Due to the high-risk population group; study staff will document any occasions where a session has had to be stopped due to participant distress or where the session is terminated because the researcher or prison staff identify a risk to the researcher. This information will be communicated to the Chair of the Trial Steering Committee within a week of it occurring. In Scotland, it will be possible to identify and document whether participants are put on a suicide risk management strategy while they are in prison. This will be monitored and any participant put onto this strategy within a week of being in contact with study staff will be flagged and reported to the Chair within 24 hours.

Dissemination

Our dissemination plan will include local, national and international avenues. The findings will be prepared for publication via open access, peer-reviewed journal articles. In addition, findings of the APPRAISE pilot trial and any plans for a future RCT will be presented to Health and Justice Teams at Scottish Government and Public Health England, the National Prisoner Healthcare Network (Scotland) and the WHO (Health in Prisons Programme Collaborating Centre). We will ensure participants have the opportunity to engage with research findings through infographics and will feedback to the participating sites. Other dissemination opportunities include presentations at meetings, workshops, national and international conferences, or at relevant organisations and events, such as Scottish Alcohol Research Network, Scottish Health Action on Alcohol Problems and Offender Health Research Network.

Patient and Public Involvement

Patient and public involvement has informed and influenced the development of the study. Representatives have included those with lived experience of imprisonment as well as those involved in the delivery of alcohol support and advice to criminal justice populations. Their expertise has informed the development of study materials and will also inform the analysis, reporting, and dissemination of the research.

For peer review only

Acknowledgements

Contributors
AH managed the overall design and inception of the study with DNB. AH and DNB were responsible for coordinating the Scottish and English study sites, respectively. VG, GW, and JB were the study co-ordinators (VG- Scotland; GW and JB- England). RP and JR are the trial statisticians. JB and ASt are the health economists. ASh, KH, PS, RS, SC have provided methodological expertise. PC, ASo, and KL have informed third party data collection. JBS, GS, JF, and SM have supported the development of intervention and participant materials. All co-authors also contributed to the revisions and final draft of the paper.

Funding:

This study is funded by the NIHR Public Health Research Programme (17/44/11). The study is funded till December 2021.

Competing Interests

None declared

Patient Consent

Not required.

Ethics Approval
The APPRAISE protocol has been approved by the East of Scotland Research Ethics Committee (19/ES/0068), National Offender Management System (2019-240), Health Board Research and Development (2019/0268), Scottish Prison Service (SPS) research and ethics committee and The University of Edinburgh's internal ethics department.

For peer review only

Table 1: Outline of APPRAISE Intervention

Element	Elements of intervention†	Enhancing Self-efficacy	Delivery method and location**
1	Preliminary discussion	Verbal persuasion	Face to Face (P)
2	Acquiring and providing information	Verbal persuasion	Face to Face (P)
3	Self-monitoring	Verbal persuasion	Face to Face (P)
4	Increasing awareness	Physiological state	Face to Face (P)
5	Situation-appraisal and appropriate coping strategies*	Vicarious experience	Face to Face (P)<br>Mobile phone (L)
6	Goal setting*	Verbal persuasion	Face to Face (P)<br>Mobile phone (L)
7	Relapse*	Performance attainment	Face to Face (P)<br>Mobile phone (L)
8	Self-evaluation/self-reinforcement*	Performance attainment	Face to Face (P)<br>Mobile phone (L)
9	Culmination	Performance attainment	Face to Face and mobile phone (L)

†(Holloway et al, 2006) *Elements 5-8 highly rated by participants in the feasibility study (PRISM-A) and will form key focus of intervention delivery by mobile phone on liberation ** P = Prison; L = Liberation

For peer review only

Table 2: APPRAISE Intervention Elements

**Element 1: Preliminary discussion**	Opening strategies
Introduction to APPRAISE study	
Introduction to APPRAISE Intervention	
Consent, confidentiality, engagement rules, trust	
**Element 2: Acquiring and providing information**	Feedback on AUDIT score
Establish perception of impact of alcohol on health and life	
Standard units of alcohol	
Recommended drinking levels	
Alcohol-related health problems	
Legal drink/drive limit	
Tips on reducing consumption	
Where to obtain information/support (prison and liberation)	
**Element 3: Self-monitoring**	Diary card – when, where, whom, type of drink, why
**Element 4: Increasing awareness**	Balance sheet – pros and cons of drinking
Physiological sensations identified	
Alternative appraisal of somatic sensations identified	
Strategies to reduce	
**Element 5: Situation-appraisal and appropriate coping strategies**	High risk situations and antecedents of over-drinking identified

For peer review only

Alternative coping strategies identified	
Coping strategies verbalised by participant	
Praise provided	
Strategies developed further through co-production	
Strategies modelled by interventionist	
Participant verbalises strategies and visualises them	
Plan for exposure/avoidance to low risk situations and high risk situations	
General control strategies: reduction in rate of drinking, sipping, low-alcohol content and alternating between soft or low-alcohol drinks	
**Element 6: Goal setting**	Setting realistic sub-goals (short-term)
Facilitating success and increasing motivation	
**Element 7: Relapse**	What happens if you relapse
What caused the relapse?	
How do I understand relapse?	
**Element 8: Self-evaluation and self-reinforcement**	Using my alcohol diary as a means of self-evaluation and self-reinforcement
Self-congratulations and rewarding my success	
What do I attribute my success to?	
**Element 9: Culmination**	Reflections and conclusions
Plans and goals reiterated and confirmed	

For peer review only

For peer review only

References

1. Coulton, S., et al., *Screening for Alcohol Use in Criminal Justice Settings: An Exploratory Study.* Alcohol and Alcoholism, 2012. **47**(4): p. 423-427.
2. Brown, N., et al., *Alcohol screening and brief intervention in a policing context: a mixed methods feasibility study.* Drug and alcohol review, 2010. **29**(6): p. 647-654.
3. Newbury-Birch, D., et al., *Alcohol Screening and Brief Interventions for Offenders in the Probation Setting (SIPS Trial): a Pragmatic Multicentre Cluster Randomized Controlled Trial.* Alcohol and Alcoholism, 2014. **49**(5): p. 540-548.
4. Newbury-Birch, D., et al., *Sloshed and sentenced: a prevalence study of alcohol use disorders among offenders in the North East of England.* International Journal of Prisoner Health, 2009. **5**(4): p. 201-211.
5. Newbury-Birch, D., et al., *A rapid systematic review of what we know about alcohol use disorders and brief interventions in the criminal justice system.* International Journal of Prisoner Health, 2016. **12**(1): p. 57-70.
6. Funk, M., et al., *A Multicountry Controlled Trial of Strategies to Promote Dissemination and Implementation of Brief Alcohol Intervention in Primary Health Care: Findings of a World Health Organization Collaborative Study.* Journal of Studies on Alcohol, 2005. **66**(3): p. 379-388.
7. Heather, N., D. Smailes, and P. Cassidy, *Development of a Readiness Ruler for use with alcohol brief interventions.* Drug and alcohol dependence, 2008. **98**(3): p. 235-240.
8. WHO, *Global status report on alcohol and health.* 2011, World Health Organisation: Geneva.
9. Revolving Doors Agency, P.C.A. and P.H. England, *Balancing Act. Addressing Health Inequalities Among People in Contact with the Criminal Justice System: A briefing for Directors of Public Health*. 2013, Revolving Doors Agency London.
10. Newbury-Birch, D., et al., *A Systematic Review of the Efficacy of Alcohol Interventions for Incarcerated People.* Alcohol and Alcoholism, 2018. **53**(4): p. 412-425.
11. Heather, N., *Spreading alcohol brief interventions from health care to non-health care settings: Is it justified?* Drugs: Education, Prevention and Policy, 2016. **23**(5): p. 359-364.
12. Ministry of Justice and P.H. England., *The impact of community-based drug and alcohol treatment on re-offending* in *Joint experimental statistical report from the Ministry of Justice and Public Health England*. 2017: London.

For peer review only - http://bmjopen.bmj.com/site/about/guidelines.xhtml

13. Newbury-Birch, D., et al., *Alcohol screening and brief interventions for offenders in the probation setting (SIPS Trial): a pragmatic multicentre cluster randomized controlled trial.* Alcohol and alcoholism, 2014. **49**(5): p. 540-548.
14. Holloway, A.S., H.E. Watson, and G. Starr, *How do we enhance problem drinkers' self-efficacy? A nurse administered intervention.* Journal of Substance Use, 2006. **11**(6): p. 375-386.
15. Holloway, A.W., H. Watson, and A. Arthur, *An evaluation of drinking refusal Self-efficacy and minimal interventions for potential problem drinkers in a general hospital setting.* Addiction, 2007. **102**: p. 1762-70.
16. Holloway, A., et al., *Alcohol Brief Interventions for male remand prisoners: Protocol for a complex intervention framework development and feasibility study.* BMJ Open, 2017.
17. Holloway, A., et al., *Alcohol brief interventions for male remand prisoners: a mixed-methods feasibility and acceptability study.* The Lancet, 2019. **394**: p. S53.
18. Heather, N., *Breaking new ground in the study and practice of alcohol brief interventions.* Drug and alcohol review, 2010. **29**(6): p. 584-588.
19. Watson, M., *A framework for development and evaluation of RCTs for complex interventions to improve health.* Int J Pharm Pract, 2000. **14**(4): p. 233-4.
20. Ministry of justice, *Proven re-offending statistics: definitions and measurement*, Ministry of Justice, Editor. 2011: London.
21. Moore, G.F., et al., *Process evaluation of complex interventions: Medical Research Council guidance.* bmj, 2015. **350**.
22. Holloway, A., et al., *Alcohol Brief Interventions for male remand prisoners: Protocol for a complex intervention framework development and feasibility study.* BMJ Open, 2017.
23. Prison Reform Trust, *Prison: the facts - Briefings, Bromley.* Prison Reform Trust. London, 2016.
24. Justice Committee, *An Inquiry into the use of Remand in Scotland*, J. Committee, Editor. 2018: Edinburgh.
25. Parker, R.A., *Overcoming Obstacles to Deriving Sample Size Calculations: Experiences of a Biostatistician.* 2020.
26. Lancaster, G.A., S. Dodd, and P.R. Williamson, *Design and analysis of pilot studies: recommendations for good practice.* Journal of evaluation in clinical practice, 2004. **10**(2): p. 307-312.
27. Teare, M.D., et al., *Sample size requirements to estimate key design parameters from external pilot randomised controlled trials: a simulation study.* Trials, 2014. **15**(1): p. 264.

For peer review only

28. Julious, S.A., *Sample size of 12 per group rule of thumb for a pilot study.* Pharmaceutical Statistics: The Journal of Applied Statistics in the Pharmaceutical Industry, 2005. **4**(4): p. 287-291.

29. Saunders, J.B., et al., *Development of the alcohol use disorders identification test (AUDIT): WHO collaborative project on early detection of persons with harmful alcohol consumption-II.* Addiction, 1993. **88**(6): p. 791-804.

30. Bandura, A., *Health promotion from the perspective of social cognitive theory.* Psychology and health, 1998. **13**(4): p. 623-649.

31. UKATT Research Team, *Cost effectiveness of treatment for alcohol problems: findings of the randomised UK alcohol treatment trial (UKATT).* Bmj, 2005. **331**(7516): p. 544.

32. Alderson, H., et al., *Supporting Looked After Children and Care Leavers In Decreasing Drugs, and alcohol (SOLID): protocol for a pilot feasibility randomised controlled trial of interventions to decrease risky substance use (drugs and alcohol) and improve mental health of looked after children and care leavers aged 12–20 years.* Pilot and feasibility studies, 2017. **3**(1): p. 25.

33. Carroll, C., et al., *A conceptual framework for implementation fidelity.* Implementation science, 2007. **2**(1): p. 40.

34. Montgomery, P., et al., *The Oxford Implementation Index: a new tool for incorporating implementation data into systematic reviews and meta-analyses.* Journal of clinical epidemiology, 2013. **66**(8): p. 874-882.

35. Lane, C., et al., *Measuring adaptations of motivational interviewing: the development and validation of the behavior change counseling index (BECCI).* Patient education and counseling, 2005. **56**(2): p. 166-173.

36. Santarius, T., et al., *Use of drains versus no drains after burr-hole evacuation of chronic subdural haematoma: a randomised controlled trial.* The Lancet, 2009. **374**(9695): p. 1067-1073.

37. Coulton, S., et al., *Effectiveness and Cost-effectiveness of Opportunistic Screening and Stepped-care Interventions for Older Alcohol Users in Primary Care.* Alcohol and Alcoholism, 2017. **52**(6): p. 655-664.

38. Deluca, P., et al., *Screening and brief interventions for adolescent alcohol use disorders presenting through emergency departments: a research programme including two RCTs.* 2020.

39. Tennant, R., et al., *The Warwick-Edinburgh mental well-being scale (WEMWBS): development and UK validation.* Health and Quality of life Outcomes, 2007. **5**(1): p. 63.

For peer review only

40. Young, R.M. and R.G. Knight, *The Drinking Expectancy Questionnaire: A revised measure of alcohol-related beliefs.* Journal of Psychopathology and Behavioral assessment, 1989. **11**(1): p. 99-112.

41. McMahon, J. and B. Jones, *The negative alcohol expectancy questionnaire.* Journal of the Association of Nurses in Substance Abuse, 1993. **12**: p. 17.

42. The EuroQol Group, *EuroQol-a new facility for the measurement of health-related quality of life.* Health policy, 1990. **16**(3): p. 199-208.

43. Bray, J.W., et al., *Measuring economic outcomes of alcohol treatment using the Economic Form 90.* Journal of studies on alcohol and drugs, 2007. **68**(2): p. 248-255.

44. Eldridge, S.M., et al., *CONSORT 2010 statement: extension to randomised pilot and feasibility trials.* bmj, 2016. **355**: p. i5239.

45. Braun, V. and V. Clarke, *Using thematic analysis in psychology.* Qualitative research in psychology, 2006. **3**(2): p. 77-101.

46. May, C. and T. Finch, *Implementation, embedding, and integration: an outline of Normalization Process Theory.* . 2009: Sociology. p. 535-54.

47. May, C.R., et al., *Evaluating complex interventions and health technologies using normalization process theory: development of a simplified approach and web-enabled toolkit.* BMC health services research, 2011. **11**(1): p. 245.

48. May, C.R., et al., *Development of a theory of implementation and integration: Normalization Process Theory.* Implementation Science, 2009. **4**(1): p. 29.

49. Murray, E., et al., *Normalisation process theory: a framework for developing, evaluating and implementing complex interventions.* BMC medicine, 2010. **8**(1): p. 63.

For peer review only

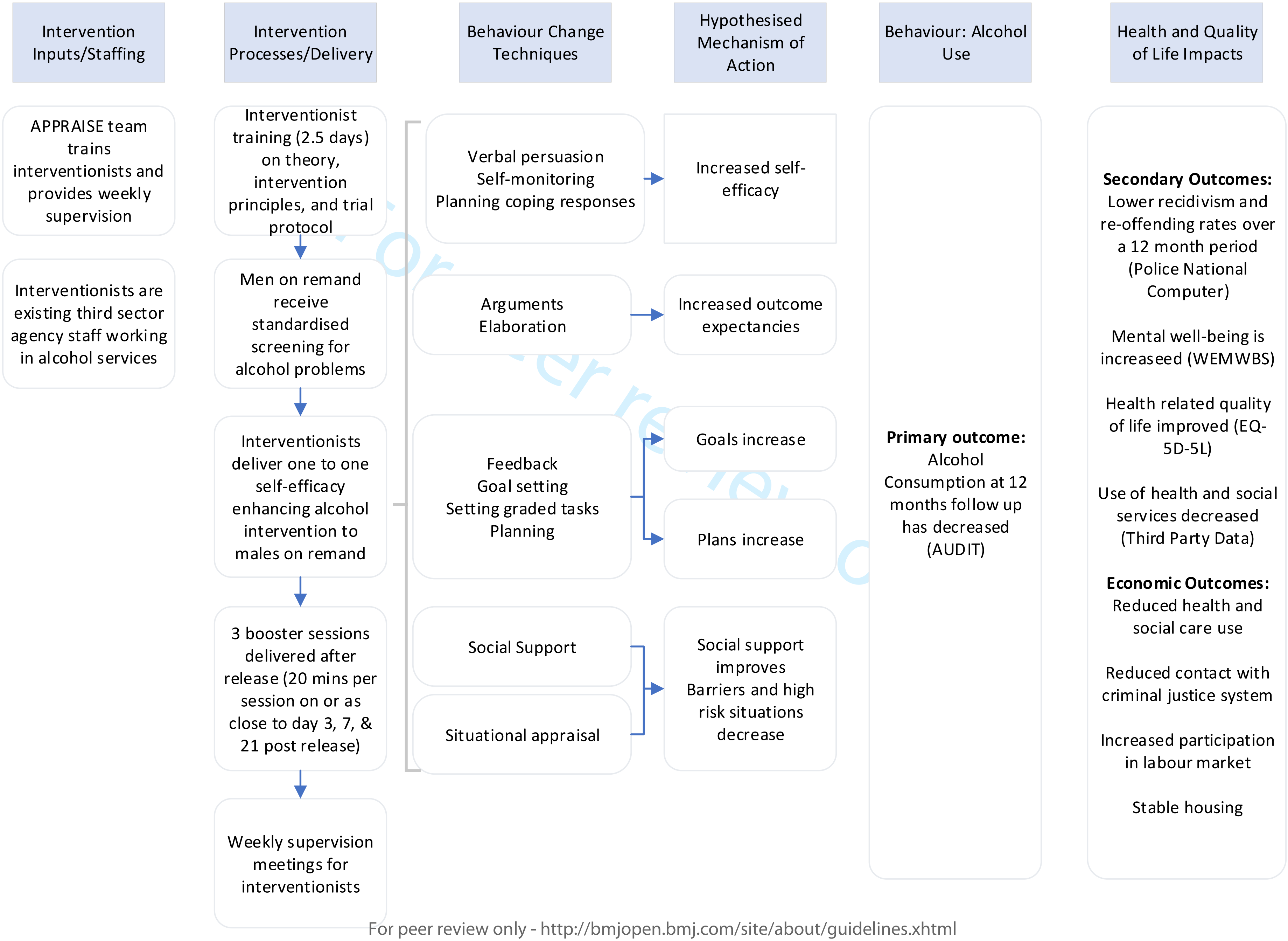
Intervention Inputs/Staffing
Intervention Processes/Delivery
Behaviour Change Techniques
Hypothesised Mechanism of Action
Behaviour: Alcohol Use
Health and Quality of Life Impacts
APPRAISE team trains interventionists and provides weekly supervision
Interventionists are existing third sector agency staff working in alcohol services
Interventionist training (2.5 days) on theory, intervention principles, and trial protocol
Men on remand receive standardised screening for alcohol problems
Interventionists deliver one to one self-efficacy enhancing alcohol intervention to males on remand
3 booster sessions delivered after release (20 mins per session on or as close to day 3, 7, & 21 post release)
Weekly supervision meetings for interventionists
Verbal persuasion
Self-monitoring
Planning coping responses
Arguments
Elaboration
Feedback
Goal setting
Setting graded tasks
Planning
Social Support
Situational appraisal
Increased self-efficacy
Increased outcome expectancies
Goals increase
Plans increase
Social support improves
Barriers and high risk situations decrease
Primary outcome: Alcohol Consumption at 12 months follow up has decreased (AUDIT)
Secondary Outcomes: Lower recidivism and re-offending rates over a 12 month period (Police National Computer)
Mental well-being is increaseed (WEMWBS)
Health related quality of life improved (EQ-5D-5L)
Use of health and social services decreased (Third Party Data)
Economic Outcomes: Reduced health and social care use
Reduced contact with criminal justice system
Increased participation in labour market
Stable housing

BMJ Open

A two-arm parallel group individually randomised Prison Pilot study of a male Remand Alcohol Intervention for Self-efficacy Enhancement: The APPRAISE Study Protocol

Journal:	*BMJ Open*
Manuscript ID	bmjopen-2020-040636.R2
Article Type:	Protocol
Date Submitted by the Author:	14-Jan-2021
Complete List of Authors:	Holloway, Aisha; University of Edinburgh, School of Health in Social Sciences<br>Guthrie, Victoria; University of Edinburgh, School of Health in Social Sciences<br>Waller, Gillian; Teesside University<br>Smith, Jamie; University of Edinburgh, School of Health in Social Sciences; Charite Universitatsmedizin Berlin<br>Boyd, Joanne; Teesside University; County Durham Drug and Alcohol Recovery Service, Centre for Change<br>Mercado, Sharon; University of Edinburgh, School of Health in Social Sciences<br>Smith, Pam; University of Edinburgh, School of Health in Social Science<br>Stenhouse, Rosie; University of Edinburgh, School of Health in Social Sciences<br>Sheikh, Aziz; University of Edinburgh, Division of Community Health Sciences<br>Parker, Richard; University of Edinburgh, Edinburgh Clinical Trials Unit<br>Stoddart, Andrew; The University Of Edinburgh, Edinburgh Health Services Research Unit<br>Conaglen, Philip; NHS Lothian<br>Coulton, Simon; University of Kent, Centre for Health Service Studies<br>Stadler, Gertraud; Charite Universitatsmedizin Berlin; University of Aberdeen<br>Hunt, Kate; University of Stirling, Institute of Social Marketing<br>Bray, Jeremy; University of North Carolina at Greensboro<br>Ferguson, Jennifer; Teesside University<br>Sondhi, Arun; Therapeutic Solutions,<br>Lynch, Kieran; Public Health England<br>Rees, Jessica; University of Edinburgh, Edinburgh Clinical Trials Unit<br>Newbury-Birch, Dorothy; Teesside University,
**Primary Subject Heading**:	Evidence based practice
Secondary Subject Heading:	Addiction, Health economics, Health services research, Nursing, Public health
Keywords:	PUBLIC HEALTH, Substance misuse < PSYCHIATRY, HEALTH

	ECONOMICS

SCHOLARONE™
Manuscripts

I, the Submitting Author has the right to grant and does grant on behalf of all authors of the Work (as defined in the below author licence), an exclusive licence and/or a non-exclusive licence for contributions from authors who are: i) UK Crown employees; ii) where BMJ has agreed a CC-BY licence shall apply, and/or iii) in accordance with the terms applicable for US Federal Government officers or employees acting as part of their official duties; on a worldwide, perpetual, irrevocable, royalty-free basis to BMJ Publishing Group Ltd ("BMJ") its licensees and where the relevant Journal is co-owned by BMJ to the co-owners of the Journal, to publish the Work in this journal and any other BMJ products and to exploit all rights, as set out in our licence.

The Submitting Author accepts and understands that any supply made under these terms is made by BMJ to the Submitting Author unless you are acting as an employee on behalf of your employer or a postgraduate student of an affiliated institution which is paying any applicable article publishing charge ("APC") for Open Access articles. Where the Submitting Author wishes to make the Work available on an Open Access basis (and intends to pay the relevant APC), the terms of reuse of such Open Access shall be governed by a Creative Commons licence – details of these licences and which Creative Commons licence will apply to this Work are set out in our licence referred to above.

Other than as permitted in any relevant BMJ Author's Self Archiving Policies, I confirm this Work has not been accepted for publication elsewhere, is not being considered for publication elsewhere and does not duplicate material already published. I confirm all authors consent to publication of this Work and authorise the granting of this licence.

For peer review only

A two-arm parallel-group individually randomised Prison Pilot study of a male Remand Alcohol Intervention for Self-efficacy Enhancement:

The APPRAISE Study Protocol

Aisha Holloway[1] (0000-0003-3070-3703),

Victoria Guthrie[1] (0000-0002-3920-2342),

Gillian Waller[2] (0000-0003-1873-3766),

Jamie B Smith[1,8] (0000-0003-0097-6102)

Joanne Boyd[2,3]

Sharon Mercado[4]

Pam Smith[1] (0000-0001-7660-0289),

Rosie Stenhouse[1] (0000-0002-1253-2544),

Aziz Sheikh[5] (0000-0001-7022-3056),

Richard Anthony Parker[6] (0000-0002-2658-5022),

Andrew Stoddart[6] (0000-0002-1958-3897),

Philip Conaglen[5] (0000-0002-8605-7045),

Simon Coulton[7] (0000-0002-7704-3274),

Gertraud Stadler[8] (0000-0002-1935-8200),

Kate Hunt [9] (0000-0002-5873-3632),

Jeremy Bray[10] (0000-0001-7712-160X),

Jennifer Ferguson[2] (0000-0002-1719-269X)

Arun Sondhi[11] (0000-0002-4748-5522),

Kieran Lynch[12] (0000-0002-4604-7838)

Jessica Rees[6] (0000-0002-1495-2470)

Dorothy Newbury-Birch[2] (0000-0003-0065-8649).

For peer review only

1) Nursing Studies, Doorway 6, Old Medical School, Teviot Place, University of Edinburgh, EH8 9AG, Scotland, UK.
2) School of Social Sciences, Humanities and Law, Teesside University, Middlesbrough, TS1 3BA, England, UK.
3) Humankind, Durham, DH1 3BQ, England, UK.
4) Patient and Public Involvement.
5) Usher Institute, Old Medical School, Teviot Place, University of Edinburgh, EH8 9AG, Scotland, UK.
6) Edinburgh Clinical Trials Unit, Usher Institute, Old Medical School,, Teviot Place, University of Edinburgh, EH8 9AG, Scotland, UK.
7) Centre for Health Service Studies, George Allen Wing, University of Kent, Canterbury, CT2 7NZ, UK.
8) Charité - Universitätsmedizin Berlin, Health & Human Sciences, Institute of Gender in Medicine (GiM)
9) Institute for Social Marketing and Health, Faculty of Heath Sciences and Sport, University of Stirling, FK9 4LA.
10) Department of Economics, University of North Carolina at Greensboro, 462 Bryan Building, P.O. Box 26170, Greensboro NC 27402-6170, U.S.A..
11) Therapeutic Solutions (Addictions) Ltd., London, UK.
12) Public Health England

For peer review only

Author Information

Aisha Holloway, BSc (Hons) Nursing, RGN, PhD, Professor of Nursing Studies at The University of Edinburgh.

Dorothy Newbury-Birch, BA (Hons), PhD, Professor of Alcohol and Public Health Research at Teesside University.

Victoria Guthrie, BSc (Hons), MSc, PhD, Senior Research Fellow at the University of Edinburgh.

Gillian Waller, BSc (Hons), MSc, PhD, Research Associate at Teesside University.

Jamie B Smith, BSc (Hons), PgDip RN, MSc, MA, Research Associate at The University of Edinburgh and Charité - Universitätsmedizin, Berlin

Joanne Boyd, Research Associate at Teesside University and Trainer for County Durham Drug and Alcohol Recovery Service.

Pam Smith, BNurs, RGN, MSc, PhD Professorial Fellow, Nursing Studies at The University of Edinburgh.

Rosie Stenhouse, PhD RMN Lecturer in Nursing Studies at The University of Edinburgh.

Aziz Sheikh, BSc, MBBS, MSc, MD, Professor of Primary Care Research and Development and Director, Usher Institute at University of Edinburgh.

Richard Parker, Bsc (Hons), MSc, Senior Statistician at Edinburgh Clinical Trials Unit, University of Edinburgh.

Andrew Stoddart, BA (Hons), MSc, Health Economist at University of Edinburgh.

Philip Conaglen, MBChB, Prof.Dip. Marketing, MSc, MFPH, Consultant in Public Health Medicine at University of Edinburgh.

For peer review only

Simon Coulton, BSc, MSc, Professor of Health Services Research at University of Kent.

Gertraud Stadler, PhD, Charité - Universitätsmedizin Berlin, Institute of Gender in Medicine (GiM), Prevention Research Group.

Kate Hunt, MA, MSc, PhD, FRSE, Professor of Behavioural Science and Health at University of Stirling.

Sharon Mercado, BA (Hons), Former Community Justice Mentor, c/o APPRAISE study team.

Jeremy Bray, BA, MA, PhD, Jefferson-Pilot Excellence, Professor, University of North Carolina at Greensboro.

Jennifer Ferguson, LLB Hons (Exempting), Graduate Tutor, PhD Researcher, Teesside University.

Arun Sondhi, BA (Hons), MSc, MBA. Researcher, Therapeutic Solutions (Addictions) Ltd.

Kieran Lynch, Programme Manager Criminal Justice, Drugs, Alcohol & Tobacco, Public Health England.

Jessica Rees, PhD, Statistician at Edinburgh Clinical Trial Unit, University of Edinburgh.

Correspondence to: Professor Aisha Holloway, BSc (Hons) Nursing, RGN, PhD

Email: aisha.holloway@ed.ac.uk

For peer review only

ABSTRACT

Introduction

The prevalence of at-risk drinking is far higher amongst those in contact with the criminal justice system (73%) than the general population (35%). However, there is little evidence on the effectiveness of alcohol brief interventions (ABIs) in reducing risky drinking amongst those in the criminal justice system, including the prison system and in particular, those on remand. Building on earlier work, APPRAISE is a pilot study designed to assess the feasibility and acceptability of an ABI, delivered to male prisoners on remand. The findings of APPRAISE should provide the information required to design a future definitive randomised controlled trial (RCT).

Methods and Analysis

APPRAISE will use mixed methods, with two linked phases, across two prisons in the UK, recruiting 180 adult men on remand: 90 from Scotland and 90 from England. Phase I will involve a 2-arm, parallel-group, individually randomised pilot study. The pilot evaluation will provide data on the likely impact of APPRAISE, which will be used to inform a future definitive multicentre RCT. Phase II will be a process evaluation assessing how the ABI has been implemented to explore the change mechanisms underpinning the ABI (Figure 1), and to assess the context within which the ABI is delivered.

Ethics and Dissemination

The APPRAISE protocol has been approved by the East of Scotland Research Ethics Committee (19/ES/0068), National Offender Management System (2019-240), Health Board Research and Development (2019/0268), Scottish Prison Service (SPS) research and ethics committee and by the University of Edinburgh's internal ethics department. The findings will be disseminated via peer-reviewed journal publications, presentations at local, national and international conferences, infographics and shared with relevant stakeholders through meetings and events.

Trial Registration Number ISRCTN 27417180

Keywords: alcohol, alcohol brief intervention (ABI), prison, remand prisoners.

Strengths and Limitations

For peer review only

- This study provides a unique opportunity to evaluate the feasibility of conducting trial based research with men on remand in prison, a frequently underrepresented group in research.
- A significant strength is that it will assess the feasibility and acceptability of the Alcohol Brief Intervention from multiple perspectives, including men on remand, prison-based intervention teams, and additional stakeholder groups.
- It will provide an opportunity to identify the operational criteria necessary for a future definitive randomised control trial.
- The unique context provides challenges for research, and the prisons' day-to-day running will influence research activities.

For peer review only

Introduction

Background/Rationale

The prevalence of at-risk drinking, which is defined as drinking at levels that harm a person's health, is far higher amongst those within the criminal justice system (73%) [1-5] than the general population (35%) [6]. In the United Kingdom (UK), between 51-83% of incarcerated people are classified as risky drinkers [7], and for those on remand in prison, the prevalence is between 62-68% [5]. Furthermore, alcohol dependence among those incarcerated (43%) is ten times higher than the general population [5]. The impacts of risky drinking are significant, resulting in significant health, economic and social burden on individuals, families and society as a whole [8]. Addressing alcohol harm in prisons, at what can be considered a 'teachable moment', could reduce the risk of re-offending, reduce costs to society and help address health inequalities [9].

There is currently robust evidence from systematic reviews and meta-analyses, indicating that Alcohol Brief Interventions (ABIs) are effective in reducing alcohol consumption amongst at-risk drinkers in healthcare settings [8, 9]. However, there is little evidence of the effectiveness of ABIs in reducing risky drinking amongst those incarcerated, particularly those on remand [10, 11], despite there being evidence in the UK that ABIs can reduce recidivism [12].

In addition, ABIs have been shown to reduce recidivism within the probation setting [13]. Our systematic review of the efficacy of psychosocial alcohol interventions for incarcerated people [10] found that interventions within prison have the potential to impact alcohol use positively; however, because of small numbers and different outcome measures, it was not possible to conduct a meta-analysis or generalise findings. Notably, none of the studies focused on men on remand, with remand referring to individuals who are un-convicted or convicted and unsentenced, held in custody awaiting trial and/or sentencing. The intervention in this proposed study builds on previous research exploring the theoretical validity of a self-efficacy enhancing A.B.I. [14, 15]. This intervention was initially tested within a pilot cluster-RCT, in a general hospital setting, and provided evidence of a potential effect [15].

In the recently completed PRISM-A study, an ABI was developed and refined within the prison setting, working with men on remand to include a synthesis of their views, with reviews of the evidence base and theoretical underpinnings [16, 17]. The findings also showed a

For peer review only - http://bmjopen.bmj.com/site/about/guidelines.xhtml

high prevalence of at-risk drinking, with 82% of participants scoring ≥8 on the Alcohol Use Disorders Test (AUDIT) [17]. This is comparable to other findings in the prison system in the UK [1-5] but is more than three times higher than primary care settings [6]. The study results indicated high levels of willingness of both staff and participants to engage with the intervention. Also, support was shown for an extended intervention, which helped men on remand to develop skills and strategies that would be useful for liberation. The feedback regarding frequency and intensity of contact, identified a preference for more than one session, including additional community-based sessions, to allow participants to put their skills into practice when alcohol is more widely available. Based on learning from interviews within the PRISM-A study, the intervention for the APPRAISE pilot study includes three post-release booster sessions.

However, there is limited evidence around the optimum timing of delivery, recommended length, content, implementation and economic benefit of an ABI in the prison setting. Furthermore, there are weaknesses within the current evidence base, with interventions not having theoretical underpinnings [11, 18]. This risks an intervention being used with a weak theoretical base, poorly specified 'active' ingredients and less likely to deliver the desired outcomes.

Following on from the positive results from the PRISM-A study, the MRC framework suggests the next step is to conduct a pilot study [19]. Therefore, the APPRAISE pilot trial has been proposed to strengthen the evidence around ABIs targeted at male remand prisoners. It will seek to assess the feasibility and acceptability of the ABI, in comparison to usual care, it will explore the potential effectiveness of the key parameters. It will also aim to provide the information required to be able to design a definitive RCT.

Aim

The APPRAISE study aims to undertake a two-arm, parallel-group, individually randomised, pilot study of a self-efficacy enhancing psychosocial alcohol intervention, for men on remand, in prison. If this pilot study is successful, it will provide the evidence to support the design of a future multicentre RCT.

The specific study objectives are:

Objective 1: To pilot the study measures and evaluation methods to assess the feasibility of conducting a multicentre, pragmatic, parallel-group, RCT.

For peer review only

1a) Is it feasible to conduct a future multicentre RCT of a self-efficacy enhancing psychosocial ABI for men on remand?

1b) Can we obtain reasonable estimates of the parameters necessary to inform the design and sample size calculation for an RCT, including standard deviations of potential continuous primary outcomes and estimates of recruitment, retention and follow-up rates?

1c) Can we determine, based on rates of missing data, whether the questionnaires are appropriate for the target population?

1d) Can we collect economic data needed for an RCT?

1e) Can we access recidivism (as measured by 'proven' reconviction rates [20] data derived from the Police National Computer (PNC) for participants)?

1f) Can we access health data from NHS data sources for participants?

Objective 2: To assess intervention fidelity

2a) What proportion of the interventions are delivered as per protocol?

2b) Is there any evidence of contamination between the two arms and/or between workers delivering the intervention?

2c) To what extent does the intervention change process variables consistent with the underpinning theory?

Objective 3: To qualitatively explore the feasibility and acceptability of the intervention and study measures to staff and participants on remand and on liberation

3) How acceptable are the trial and intervention procedures (including context and any barriers and facilitators) to the key stakeholders: men on remand in prison and on liberation; prison staff (including healthcare staff); commissioners; policymakers and third sector partners?

Objective 4: To assess whether operational progression criteria for conducting a future definitive RCT are met across trial arms and study sites and if so, develop a protocol for an RCT. *Operational progression criterion are based on previous research results [3]*

4a) Do the two prisons invited to the study agree to take part?

4b) Based on knowledge from previous data, do at least 90 eligible participants consent to take part and be randomised?

4c) Do at least 70% of participants allocated to the intervention condition, go on to receive at least one intervention session?

For peer review only

4d) Do at least 60% of participants take part in the follow-up assessments at 12 months across trial arms and study sites?

Methods and Analysis

Study Design

The study aligns to the MRC framework [19], using mixed methods within two linked phases conducted across two sites.

Phase I will involve a two-arm, parallel-group, individually randomised pilot study (Objectives 1 and 4). The pilot evaluation will provide data on feasibility and an assessment on the likely impact of the ABI to inform the feasibility and design of a future RCT.

Phase II will comprise a process evaluation (Objectives 2 and 3), drawing on the MRC process evaluation framework to guide the planning and design of the evaluation [21]. The aim of the process evaluation is: to assess how the ABI is implemented; to undertake a preliminary exploration of the change mechanisms underpinning the intervention (Figure 1), and assess the delivery context of the ABI.

Participants and Setting

Identification of Study Participants

A suitable process of participant identification was established and found to be successful in the PRISM-A study [17, 22]. The prison induction team (consisting of prison officers and peer prisoners) will provide potential participants with a verbal account of the study and a Participant Information Sheet (PIS) during their prison induction. Researchers will liaise with relevant prison officers each week to identify potential participants. Potential participants will be contacted via prison staff; those who are willing will meet with the researcher in an area of the prison where they can discuss issues in private, without risk of being overheard, and without risk to the interviewer or disruption to the running of the prison. The researcher will review the PIS with the participant, provide a written and verbal description of APPRAISE, answer any questions, and invite him to consider participating. The researcher will then obtain written consent from willing participants. Those who do not wish to participate will receive no further interaction with

For peer review only

the research staff. Data collection began on 1st December 2019 and will finish on 31st August 2021.

Sample Size

180 adult men (18 years and over) on remand will be recruited from two sites: one within the Scottish Prison Service (SPS); Edinburgh (n=90) and the second from Her Majesty's Prison and Probation Service (HMPS); Durham, England (n=90). These sites were purposively selected following PRISM-A, as they offer geographical, socio-economic and ethnic diversity, along with differing procedures and structures [16]. Both prisons provide alcohol services via external agencies who engage with people in custody and following release. Prisons have varying induction procedures, communication mechanisms and processes, and available, secure space to engage participants. Understanding the different approaches and dynamics will be important when designing a future RCT.

The target population is men on remand in prison, who have been in prison for three months or less. The average time on remand is approximately nine weeks in England [23] and four weeks in Scotland [24]. We estimate that approximately 50% of participants will be released, while the rest will remain incarcerated; leaving 45 participants per study arm (90 in total) across the two sites.

This sample size was calculated to take account of the following factors [25]:

1. It will enable calculations of two-sided 95% confidence intervals (CIs) around proportions recruited, released, and drop-out in each study arm with half-widths of less than 0.15.

2. It exceeds the 30 per group recommendation of Lancaster et al. [26] and 35 participants per group recommendation of Teare et al. [27] for estimating key unknown design parameters, e.g. standard deviation with sufficient precision with a continuous primary outcome.

3. It will ensure that within each arm, across each site, we satisfy the minimum 12 per group' rule of thumb' outlined by Julious [28] for pilot trials.

Participant Eligibility Criteria

For peer review only

The following criteria were used in PRISM-A and were found to be appropriate.

Inclusion Criteria

Participants will be included in APPRAISE if they meet the following inclusion criteria:

- Informed consent given
- Men aged 18 years or over
- Have been in prison for three months or less on the current charge
- Score 8 or more on the AUDIT [29]
- Detained in either of the study sites within the SPS or HMPS.

Exclusion Criteria

Participants will be excluded from APPRAISE if they meet any of the following criteria:

- Previously recruited to APPRAISE
- Unable to give informed consent or deemed incompetent/unable to make an informed decision regarding consent
- Identified as a risk to self and/ or others by prison staff
- Judged to be under the influence of an illicit substance by prison or research staff.
- Currently taking Disulfiram (frequently referred to as Antabuse).
- On a segregative rule (under prison rules)
- Not able to understand the documents (English language) or agree to the researcher aiding their understanding.

Informed Consent

All participants will be required to provide informed consent before taking part; their decision will be entirely voluntary and based on a clear understanding of what is involved. To ensure this, all participants will receive a verbal explanation of the study, in addition to a PIS. The verbal explanation will be given by the researcher and will cover all elements in the PIS and the consent form. Each willing participant will sign a consent form to ensure that informed consent has been obtained. Each participant will receive a copy of the form, and a copy will be filed in the Investigator Site File (ISF). All participants will be given sufficient time to consider the information provided, ask questions, and clarify anything they do not understand. Participants will be

For peer review only

reassured that they can withdraw their consent at any time, without loss of benefits to which they otherwise would be entitled.

Ethics and Dissemination

The APPRAISE protocol has been approved by the East of Scotland Research Ethics Committee (19/ES/0068), National Offender Management System (2019-240), Health Board Research and Development (2019/0268), Scottish Prison Service (SPS) research and ethics committee and by the University of Edinburgh's internal ethics department. The findings will be disseminated via peer-reviewed journal publications, presentations at local, national and international conferences, infographics and shared with relevant stakeholders through meetings and events.

The APPRAISE Intervention

The ABI has four sessions to be offered to all participants in the intervention arm. It will be delivered by existing staff at Change Grow Live (CGL) who are currently engaged in alcohol service delivery in the prison and community setting. The ABI comprises of nine elements (See Table 1 and 2) and will be delivered in 4 steps: Step 1 is 1 x 40-minute, face-to-face session, covering nine elements, delivered by a trained CGL staff member within the prison. Steps 2, 3 and 4 are 20-minute sessions, by phone, on or as close to day 3, 7, and 21 post-liberation. Exact days will be recorded to inform the process evaluation. The post-liberation sessions will include elements 1 (preliminary discussion), 5 (situation-appraisal), 6 (goal setting), 7 (relapse), 8 (self-evaluation/self-reinforcement and 9 (culmination).

Self-efficacy derives from Social Cognitive Theory and has been identified as an important determinant of health behaviour and health behaviour change [30]. The four primary sources of self-efficacy information (that can be targeted through interventions) are mastery experience; vicarious experience; verbal persuasion; and physiological state [14, 15, 30]. The ABI is designed to increase self-efficacy – through the development of self-regulatory skills, self-management and self-belief, thus enabling an individual to address their alcohol consumption. Liberation then offers participants the ability to develop and build upon these skills and self-belief through success and mastery, with their efforts leading to the adoption and maintenance of reduced alcohol consumption [14, 30]. Reducing alcohol consumption can provide a sense of achievement and success, with the overall effects of such an intervention a likely increase in the men's level of self-efficacy [14].

For peer review only - http://bmjopen.bmj.com/site/about/guidelines.xhtml

Staff Training

Two members of CGL staff at each site will be trained to deliver the ABI to participants (intervention), and the remaining staff will have no training (control). This method seeks to minimise contamination and has been used in other similar studies [31, 32]. The possibility of residual contamination and the practicality of allocating staff according to randomisation will be explored within the process evaluation to inform the future RCT. Weekly de-briefs with interventionists delivering the APPRAISE ABI will also enable the exploration of possible contamination.

Intervention Delivery

The ABI has four sessions to be offered to all participants in the intervention arm. Understanding what has been delivered and how is a key component of understanding the ABI implementation [33, 34]. As part of the process evaluation to assess implementation, measures of fidelity (quality) and dose (quantity) will be recorded and analysed. To capture fidelity, we will digitally audio record 20% of intervention sessions and code these, using the Behaviour Change Counselling Index (BECCI) [35], to assess the extent to which the essential theoretical elements of the ABI were delivered as intended. To capture dose, quantitative data from participant study records and logs will be obtained to assess the proportion of interventions offered and delivered successfully. The number of intervention sessions offered, delivered, and the length of each session will be recorded as will reasons for any unsuccessful delivery. Interviews with all implementers and 32 purposively selected participants will also be conducted at each site to identify how acceptable the trial intervention and procedures were.

Control Condition (Care as Usual)

The control condition will include participants receiving care as they usually would, within the existing service provision. What constitutes care as usual, will vary between participants as some participants may access no support. In contrast, others may attend individual or group-based support sessions with CGL and/or other providers whilst in prison or their local communities. It should be noted that participants in the intervention condition will also have access to care as usual services. Service utilisation will be recorded for all participants.

For peer review only - http://bmjopen.bmj.com/site/about/guidelines.xhtml

Data Collection

Baseline Assessment

Following appropriate consent procedures, baseline (TP0) data will be collected via researcher-led completion of questionnaires, and this will take approximately 30 minutes. Immediately after, participants will be randomised to the control or intervention arm and informed of their allocation. Participants randomised to the intervention arm will be provided with a face-to-face appointment to meet with their interventionist. This is the process used by the current providers (CGL) when engaging with individuals in the prison setting. The three telephone sessions will be delivered following release from prison by one of the trained interventionists. In addition to the outcome measures (described below), data on age, gender, marital status, educational status, and contact details will be collected.

Blinding

Due to restrictions regarding electronic equipment and mobile phone use by researchers whilst in prison, a randomisation system, such as an Interactive Voice Response telephone system or one accessed over the web, cannot be used. Allocation will be conducted at participant level using random permuted blocks of variable size [3, 5, 6] stratified by site using sealed envelopes, based on a predetermined random number allocation carried out by Edinburgh Clinical Trials Unit (ECTU) [28, 36].

Researchers will not be involved in delivering the active and control interventions but will be aware of the study allocation of participants as the trial progresses. Allocation concealment will be used whereby neither the person delivering the interventions nor the participant will be aware of the study allocation until they are irrevocably entered into the trial. Both the trial statistician (RP) and health economist (JB) will be blind to group allocation and will only have details of study participants by study number.

Follow-up

Follow-up assessments will be conducted at 6 (TP1) and 12 months (TP2) post recruitment. The original aim was for these to be conducted by the researcher either in person or by telephone. In light of COVID-19 restrictions, follow-up assessments have also been adapted to enable self-

For peer review only - http://bmjopen.bmj.com/site/about/guidelines.xhtml

completion using hard copies or electronic versions. Where participants are identified as being incarcerated at follow-up, arrangements with the relevant prison will be made to undertake the follow-up. Attempts to contact participants will be multi-faceted, including telephone calls, text messages, and e-mails, based on participants' preferred method of contact, at varying times and days of the week. We will record the number of participants invited to participate; responded; were eligible; recruited; transferred; subsequently sentenced; liberated, and lost to follow-up. The number who returned to prison during the 12 months and who completed the trial will also be recorded.

Outcomes

Primary outcome measure

The proposed primary outcome measure is alcohol consumed, in units where one unit equals 8g or 10ml of ethanol, per week will be derived from the frequency and quantity of alcohol consumption questions in the extended version of AUDIT using a formula developed and employed in a number of randomised controlled trials of interventions for alcohol consumption [37, 38]. A score of 8+ is referred to as a 'positive screen' and indicates drinking at hazardous (score of 8–15), harmful (score of 16-19), or probable dependent level (score of 20+). A score of 8 or more out of a possible 40 on the AUDIT has an established sensitivity of 92% and specificity of 94% [29] to detect those at-risk of harm from alcohol use. The AUDIT will be administered at TP0, TP1 and TP2.

Self-report secondary outcomes measures

Secondary outcome measures will be completed at TP0, TP1 and TP2.

The Warwick-Edinburgh Mental Well-being scale (WEMWBS) will be used to assess mental well-being [39]. This tool uses a 5-point Likert scale, which gives a score of one to five per question, giving a minimum score of 14 and maximum score of 70. A higher WEMWBS score indicates a higher level of mental well-being [39].

Readiness to change will be measured using the Readiness to Change Ruler, which measures readiness to change drinking behaviour [7].

For peer review only

Self-reported alcohol self-efficacy will be measured using the Drinking Refusal Self-Efficacy Questionnaire – revised (DRSEQ-R) [40] and alcohol expectancy, using the Negative Alcohol Expectancy Questionnaire (NAEQ) [41].

The Euroqol (EQ-5D -5L) will be used to measure health-related quality of life and is being scoped for potential future use to generate Quality Adjusted Life Years [42]. A bespoke public sector service use questionnaire will be adapted based on the economic form 90 [43] to determine public sector costs in the domains of health and social care, criminal justice system, unemployment, and welfare.

Other secondary outcome measures

Attempts will be made to access data from the Police National Computer (PNC) to collect individual-level data on recidivism rates as measured by 'proven' reconviction rates [20] over 12 months, since TP0. The number of participants incarcerated at TP1 and TP2 will also be recorded.

Individual-level data relating to health and social care use will also be sought. If access is possible, data derived from Community Health Index (CHI)/ NHS number at TP3 will be used to validate the bespoke public sector service use questionnaire. Furthermore, data will be collected on the time spent by researchers and practitioners on APPRAISE to inform the resources needed for a definitive RCT.

Mechanisms of impact

An intervention logic model (Figure 1) informed by social cognitive theory has been developed. The underlying causal mechanisms will be examined, to provide increased understanding of how the intervention influences change through the quantitative assessment of key behavioural markers (mediators) of change, i.e. self-reported self-efficacy, using the Drinking Refusal Self-Efficacy Questionnaire- Revised (DRSEQ-R) [40] and alcohol expectancy, using the Negative Alcohol Expectancy Questionnaire (NAEQ) [41]. These behavioural markers of change will be recorded at TP0, TP1 and TP2. An exploratory prognostic analysis will be undertaken to explore the nature of change within these domains and their relationship to the primary outcome. The relationship between alcohol consumption, receipt of the intervention and re-offending will

For peer review only

also be assessed. In addition, we will be interviewing 32 men who took part in the study (16 at each site), eight intervention staff from CGL, and 10 additional stakeholders. The qualitative exploration of participant responses to and interactions with the intervention will be undertaken, to identify unanticipated pathways and consequences at 12 months follow up.

Data Analysis

The progress of participants through the APPRAISE pilot trial will be presented in accordance with the CONSORT guidelines [44] to allow descriptions of key parameters required for a future RCT; eligibility rates, consent, adherence, retention at follow-up and data completeness of outcome measures.

The statistical analyses will be primarily descriptive, providing an estimate of the proportion of those eligible, consenting, adhering to the intervention and retention rates at 6 and 12 months. These parameters will inform the power calculations for a future RCT and confirm or identify the need for modifications in relation to other aspects of the trial design, particularly the acceptability of study processes and outcome measures to participants and staff. Data about the flow of participants through the APPRAISE pilot trial will include numbers screened, the prevalence of the target condition, numbers providing contact details, numbers eligible and willing to consent and numbers followed up successfully at TP1 and TP2. In addition, data completeness for each of the instruments and any potential bias in the follow-up data will be ascertained.

Analysis of the primary outcome will also provide estimates of the variance within and between groups for exploration of sample size calculations for a definitive RCT. Furthermore, we will calculate the variance of all primary and secondary outcome measures within groups to explore their utility in a definitive study. If access to PNC data is possible, proven reconviction rates will be measured by the number of offences committed by each treated participant against the number committed in the comparison group. Time-to-event analysis will also explore differences in offending patterns.

Calculation of between trial-arm differences (with 95% confidence intervals) will indicate the likely effect sizes that will be observed in a future definitive trial.

For peer review only - http://bmjopen.bmj.com/site/about/guidelines.xhtml

For the primary outcome analysis, the mean difference (with 95% confidence interval) in alcohol units consumed between the two randomisation groups will be calculated, both overall and stratified by site. A complete cases analysis will be used, but a sensitivity analysis will also be incorporated to explore the effects of missing data. This will involve using multiple imputations for the primary outcome in a supplementary analysis, provided that the valid sample size is sufficient to support this. In addition, we will add and subtract one-unit differences (alcohol units) to the missing data as part of a sensitivity analysis to explore how the 95% CIs change depending on our assumptions about the missing data (bearing in mind that the imputed data cannot be less than zero). This is similar to the sensitivity analysis method suggested in White et al. (2011) which involves determining how large an amount can *"be added to or subtracted from the imputed data without changing the clinical interpretation of the trial".* We will adopt a similar approach to determine how sensitive the conclusions of the APPRAISE pilot trial (and the decision about whether to proceed with an RCT) are to our assumptions about missing data.

A Statistical Analysis Plan will be written and finalised prior to analysis.

Health Economic Evaluation

Full details of all health economic analyses will be specified in a Health Economic Analysis Plan, authored by the trial health economist(s), and signed off by the PI prior to analysis.

In brief, this will scope the feasibility of conducting a future health economic evaluation alongside any future trial. A bespoke survey of public sector service use will be adapted from the Economic Form 90 [43] and tested in the patient population. We will also scope the potential for using the EQ-5D-5L relative to other health outcome measures being used in the trial. The rates of response and completeness of questionnaires will be reported in each case. A dry run costing/QALY generation exercise will be undertaken to identify any practical issues in using these instruments. The dry run costing exercise will include a detailed scoping of the cost elements relating directly to the introduction and running of the intervention through consultation with key staff involved in the implementation. Finally, the potential for longer-term modelling of outcomes will be scoped through consultation with experts and non-systematic searches of the literature for similar work or anticipated parameters. These will form the basis for a short report to aid in developing a future protocol for a follow-up trial.

For peer review only

Additional Analyses

All interview data will be transcribed and thematic analysis undertaken in order to fulfil the objectives 1d, 2c, 3a. Braun and Clarke's thematic analysis framework will guide this, as it is congruent with the study's theoretical perspective [45]. To facilitate an understanding of each stakeholder group's perspective, the data will initially be analysed within group. The rationale for undertaking the analysis within each group is to enable the targeting of remedial actions within a future RCT. Themes running across the data, in relation to each question, will then be collated, providing a multi-perspective understanding of the stakeholder experience for each element of the intervention/study.

The qualitative analysis will provide evidence to verify the feasibility and acceptability of the ABI to participants, specifically elements 5-8. It will also inform the process evaluation in Phase 2 to better understand and explain the social processes of the intervention, through the lens of implementation theory, Normalisation Process Theory [46-49].

Monitoring

An APPRAISE Trial Steering Committee (TSC) has been set up to monitor the implementation, to provide an independent assessment of the data analysis and to determine if a future trial is merited.

The TSC has the following objectives:

- Provide supervision of the trial on behalf of the sponsor and funder and ensure it is conducted to rigorous standards.
- Monitor the ongoing progress and adherence to protocol.
- Consider new information of relevance to the research question.
- Provide advice, through the chair, to the chief investigator and funder on all appropriate aspects.
- Provide evidence to support extension requests.

In addition to the TSC, the chair plus three TSC members (specialising in health economics, statistics, and qualitative methodology and analysis) have formed a subcommittee responsible for data monitoring and ethics (DMEC).

For peer review only

The DMEC subcommittee has the following objectives:

- To monitor the data and recommend the TSC on whether there are any ethical or safety reasons why the trial should not continue.
- To ensure that the safety, rights, and well-being of participants remains paramount.

The sub-committee will also consider the need for any interim analysis and are the only body who, if necessary, will have access to the unblinded data.

Data Management

Data from both study sites will be anonymised, and individuals' data will only be identifiable by their unique screening number. All of the hard copy data from the Durham site will be sent to the coordinating site, The University of Edinburgh, by secure courier, where all of the data will then be stored in a locked filing cabinet, with restricted access. Personal or sensitive data will be transported separately between sites securely, using password protected files. No personal data will be transferred outside the UK, or stored or collected on computer servers outside the U.K.

Audio files will be uploaded securely to a password-protected site by the researchers, where the approved transcription company will be able to access the files. Transcribed files will be returned to the researchers using the same secure platform and downloaded and stored securely in folders on the University of Edinburgh's password-protected, server. Following a quality check to ensure the transcribed files' accuracy, all audio recordings will be securely destroyed.

Confidentiality and Data Protection

All study staff will comply with the requirements of the appropriate data protection legislation (including the General Data Protection Regulation and Data Protection Act) concerning the collection, storage, processing and disclosure of personal information. All evaluation forms, reports, and other records will maintain participant confidentiality and only be identifiable via a unique participant identification number. All written records will be kept in secure storage, and computers used to collate the data will be password protected. Published results will not contain any personal data to ensure that individuals cannot be identified. Personal data will be stored for 24 months after the study completion, and following this will be securely destroyed.

For peer review only

Ethical Considerations

Ethical approval has been obtained from the East of Scotland NHS Research Ethics Committee, the Scottish Prisons Service Research Ethics Committee, Her Majesty's Prison and Probation Service and the University of Edinburgh School of Health in Social Science Research Ethics Committee.

Due to the high-risk population group; study staff will document any occasions where a session has had to be stopped due to participant distress or where the session is terminated because the researcher or prison staff identify a risk to the researcher. This information will be communicated to the Chair of the Trial Steering Committee within a week of it occurring. In Scotland, it will be possible to identify and document whether participants are put on a suicide risk management strategy while they are in prison. This will be monitored and any participant put onto this strategy within a week of being in contact with study staff will be flagged and reported to the Chair within 24 hours.

Dissemination

Our dissemination plan will include local, national and international avenues. The findings will be prepared for publication via open access, peer-reviewed journal articles. Also, the APPRAISE pilot trial findings and any plans for a future RCT will be presented to Health and Justice Teams at Scottish Government and Public Health England, the National Prisoner Healthcare Network (Scotland) and the WHO (Health in Prisons Programme Collaborating Centre). We will ensure participants can engage with research findings through infographics and will feedback to the participating sites. Other dissemination opportunities include presentations at meetings, workshops, national and international conferences, or at relevant organisations and events, such as Scottish Alcohol Research Network, Scottish Health Action on Alcohol Problems and Offender Health Research Network.

Patient and Public Involvement

Patient and public involvement has informed and influenced the development of the study. Representatives have included those with lived experience of imprisonment and those involved in the delivery of alcohol support and advice to criminal justice populations. Their expertise has

For peer review only - http://bmjopen.bmj.com/site/about/guidelines.xhtml

informed the development of study materials and will also inform the analysis, reporting, and dissemination of the research.

For peer review only

For peer review only - http://bmjopen.bmj.com/site/about/guidelines.xhtml

Acknowledgements

Contributors

AH managed the overall design and inception of the study with DNB. AH and DNB were responsible for coordinating the Scottish and English study sites, respectively. VG, GW, and JB were the study co-ordinators (VG- Scotland; GW and JB- England). RP and JR are the trial statisticians. JB and ASt are the health economists. ASh, KH, PS, RS, SC have provided methodological expertise. PC, ASo, and KL have informed third party data collection. JBS, GS, JF, and SM have supported the development of intervention and participant materials. All co-authors also contributed to the revisions and final draft of the paper.

Funding:

This study is funded by the NIHR Public Health Research Programme (17/44/11).

Competing Interests

None declared

Patient Consent

Not required.

Ethics Approval

The APPRAISE protocol has been approved by the East of Scotland Research Ethics Committee (19/ES/0068), National Offender Management System (2019-240), Health Board Research and Development (2019/0268), Scottish Prison Service (SPS) research and ethics committee and The University of Edinburgh's internal ethics department.

For peer review only

Table 1: Outline of APPRAISE Intervention

Element	Elements of intervention†	Enhancing Self-efficacy	Delivery method and location**
1	Preliminary discussion	Verbal persuasion	Face to Face (P) Mobile phone (L)
2	Acquiring and providing information	Verbal persuasion	Face to Face (P)
3	Self-monitoring	Verbal persuasion	Face to Face (P)
4	Increasing awareness	Physiological state	Face to Face (P)
5	Situation-appraisal and appropriate coping strategies*	Vicarious experience	Face to Face (P) Mobile phone (L)
6	Goal setting*	Verbal persuasion	Face to Face (P) Mobile phone (L)
7	Relapse*	Performance attainment	Face to Face (P) Mobile phone (L)
8	Self-evaluation/self-reinforcement*	Performance attainment	Face to Face (P) Mobile phone (L)
9	Culmination	Performance attainment	Face to Face and mobile phone (L)

†(Holloway et al, 2006) *Elements 5-8 highly rated by participants in the feasibility study (PRISM-A) and will form key focus of intervention delivery by mobile phone on liberation ** P = Prison; L = Liberation

For peer review only - http://bmjopen.bmj.com/site/about/guidelines.xhtml

Table 2: APPRAISE Intervention Elements

**Element 1: Preliminary discussion**	Opening strategies
Introduction to APPRAISE study	
Introduction to APPRAISE Intervention	
Consent, confidentiality, engagement rules, trust	
**Element 2: Acquiring and providing information**	Feedback on AUDIT score
Establish perception of impact of alcohol on health and life	
Standard units of alcohol	
Recommended drinking levels	
Alcohol-related health problems	
Legal drink/drive limit	
Tips on reducing consumption	
Where to obtain information/support (prison and liberation)	
**Element 3: Self-monitoring**	Diary card – when, where, whom, type of drink, why
**Element 4: Increasing awareness**	Balance sheet – pros and cons of drinking
Physiological sensations identified	
Alternative appraisal of somatic sensations identified	
Strategies to reduce	
**Element 5: Situation-appraisal and appropriate coping strategies**	High risk situations and antecedents of over-drinking identified

For peer review only

Alternative coping strategies identified	
Coping strategies verbalised by participant	
Praise provided	
Strategies developed further through co-production	
Strategies modelled by interventionist	
Participant verbalises strategies and visualises them	
Plan for exposure/avoidance to low risk situations and high risk situations	
General control strategies: reduction in rate of drinking, sipping, low-alcohol content and alternating between soft or low-alcohol drinks	
**Element 6: Goal setting**	Setting realistic sub-goals (short-term)
Facilitating success and increasing motivation	
**Element 7: Relapse**	What happens if you relapse
What caused the relapse?	
How do I understand relapse?	
**Element 8: Self-evaluation and self-reinforcement**	Using my alcohol diary as a means of self-evaluation and self-reinforcement
Self-congratulations and rewarding my success	
What do I attribute my success to?	
**Element 9: Culmination**	Reflections and conclusions
Plans and goals reiterated and confirmed	

For peer review only

References

1. Coulton, S., et al., *Screening for Alcohol Use in Criminal Justice Settings: An Exploratory Study.* Alcohol and Alcoholism, 2012. **47**(4): p. 423-427.
2. Brown, N., et al., *Alcohol screening and brief intervention in a policing context: a mixed methods feasibility study.* Drug and alcohol review, 2010. **29**(6): p. 647-654.
3. Newbury-Birch, D., et al., *Alcohol Screening and Brief Interventions for Offenders in the Probation Setting (SIPS Trial): a Pragmatic Multicentre Cluster Randomized Controlled Trial.* Alcohol and Alcoholism, 2014. **49**(5): p. 540-548.
4. Newbury-Birch, D., et al., *Sloshed and sentenced: a prevalence study of alcohol use disorders among offenders in the North East of England.* International Journal of Prisoner Health, 2009. **5**(4): p. 201-211.
5. Newbury-Birch, D., et al., *A rapid systematic review of what we know about alcohol use disorders and brief interventions in the criminal justice system.* International Journal of Prisoner Health, 2016. **12**(1): p. 57-70.
6. Funk, M., et al., *A Multicountry Controlled Trial of Strategies to Promote Dissemination and Implementation of Brief Alcohol Intervention in Primary Health Care: Findings of a World Health Organization Collaborative Study.* Journal of Studies on Alcohol, 2005. **66**(3): p. 379-388.
7. Heather, N., D. Smailes, and P. Cassidy, *Development of a Readiness Ruler for use with alcohol brief interventions.* Drug and alcohol dependence, 2008. **98**(3): p. 235-240.
8. WHO, *Global status report on alcohol and health*. 2011, World Health Organisation: Geneva.
9. Revolving Doors Agency, P.C.A. and P.H. England, *Balancing Act. Addressing Health Inequalities Among People in Contact with the Criminal Justice System: A briefing for Directors of Public Health*. 2013, Revolving Doors Agency London.
10. Newbury-Birch, D., et al., *A Systematic Review of the Efficacy of Alcohol Interventions for Incarcerated People.* Alcohol and Alcoholism, 2018. **53**(4): p. 412-425.
11. Heather, N., *Spreading alcohol brief interventions from health care to non-health care settings: Is it justified?* Drugs: Education, Prevention and Policy, 2016. **23**(5): p. 359-364.
12. Ministry of Justice and P.H. England., *The impact of community-based drug and alcohol treatment on re-offending* in *Joint experimental statistical report from the Ministry of Justice and Public Health England*. 2017: London.

For peer review only - http://bmjopen.bmj.com/site/about/guidelines.xhtml

13. Newbury-Birch, D., et al., *Alcohol screening and brief interventions for offenders in the probation setting (SIPS Trial): a pragmatic multicentre cluster randomized controlled trial.* Alcohol and alcoholism, 2014. **49**(5): p. 540-548.
14. Holloway, A.S., H.E. Watson, and G. Starr, *How do we enhance problem drinkers' self-efficacy? A nurse administered intervention.* Journal of Substance Use, 2006. **11**(6): p. 375-386.
15. Holloway, A.W., H. Watson, and A. Arthur, *An evaluation of drinking refusal Self-efficacy and minimal interventions for potential problem drinkers in a general hospital setting.* Addiction, 2007. **102**: p. 1762-70.
16. Holloway, A., et al., *Alcohol Brief Interventions for male remand prisoners: Protocol for a complex intervention framework development and feasibility study.* BMJ Open, 2017.
17. Holloway, A., et al., *Alcohol brief interventions for male remand prisoners: a mixed-methods feasibility and acceptability study.* The Lancet, 2019. **394**: p. S53.
18. Heather, N., *Breaking new ground in the study and practice of alcohol brief interventions.* Drug and alcohol review, 2010. **29**(6): p. 584-588.
19. Watson, M., *A framework for development and evaluation of RCTs for complex interventions to improve health.* Int J Pharm Pract, 2000. **14**(4): p. 233-4.
20. Ministry of justice, *Proven re-offending statistics: definitions and measurement*, Ministry of Justice, Editor. 2011: London.
21. Moore, G.F., et al., *Process evaluation of complex interventions: Medical Research Council guidance.* bmj, 2015. **350**.
22. Holloway, A., et al., *Alcohol Brief Interventions for male remand prisoners: Protocol for a complex intervention framework development and feasibility study.* BMJ Open, 2017.
23. Prison Reform Trust, *Prison: the facts - Briefings, Bromley.* Prison Reform Trust. London, 2016.
24. Justice Committee, *An Inquiry into the use of Remand in Scotland*, J. Committee, Editor. 2018: Edinburgh.
25. Parker, R.A., *Overcoming Obstacles to Deriving Sample Size Calculations: Experiences of a Biostatistician.* 2020.
26. Lancaster, G.A., S. Dodd, and P.R. Williamson, *Design and analysis of pilot studies: recommendations for good practice.* Journal of evaluation in clinical practice, 2004. **10**(2): p. 307-312.
27. Teare, M.D., et al., *Sample size requirements to estimate key design parameters from external pilot randomised controlled trials: a simulation study.* Trials, 2014. **15**(1): p. 264.

For peer review only

28. Julious, S.A., *Sample size of 12 per group rule of thumb for a pilot study.* Pharmaceutical Statistics: The Journal of Applied Statistics in the Pharmaceutical Industry, 2005. **4**(4): p. 287-291.
29. Saunders, J.B., et al., *Development of the alcohol use disorders identification test (AUDIT): WHO collaborative project on early detection of persons with harmful alcohol consumption-II.* Addiction, 1993. **88**(6): p. 791-804.
30. Bandura, A., *Health promotion from the perspective of social cognitive theory.* Psychology and health, 1998. **13**(4): p. 623-649.
31. UKATT Research Team, *Cost effectiveness of treatment for alcohol problems: findings of the randomised UK alcohol treatment trial (UKATT).* Bmj, 2005. **331**(7516): p. 544.
32. Alderson, H., et al., *Supporting Looked After Children and Care Leavers In Decreasing Drugs, and alcohol (SOLID): protocol for a pilot feasibility randomised controlled trial of interventions to decrease risky substance use (drugs and alcohol) and improve mental health of looked after children and care leavers aged 12–20 years.* Pilot and feasibility studies, 2017. **3**(1): p. 25.
33. Carroll, C., et al., *A conceptual framework for implementation fidelity.* Implementation science, 2007. **2**(1): p. 40.
34. Montgomery, P., et al., *The Oxford Implementation Index: a new tool for incorporating implementation data into systematic reviews and meta-analyses.* Journal of clinical epidemiology, 2013. **66**(8): p. 874-882.
35. Lane, C., et al., *Measuring adaptations of motivational interviewing: the development and validation of the behavior change counseling index (BECCI).* Patient education and counseling, 2005. **56**(2): p. 166-173.
36. Santarius, T., et al., *Use of drains versus no drains after burr-hole evacuation of chronic subdural haematoma: a randomised controlled trial.* The Lancet, 2009. **374**(9695): p. 1067-1073.
37. Coulton, S., et al., *Effectiveness and Cost-effectiveness of Opportunistic Screening and Stepped-care Interventions for Older Alcohol Users in Primary Care.* Alcohol and Alcoholism, 2017. **52**(6): p. 655-664.
38. Deluca, P., et al., *Screening and brief interventions for adolescent alcohol use disorders presenting through emergency departments: a research programme including two RCTs.* 2020.
39. Tennant, R., et al., *The Warwick-Edinburgh mental well-being scale (WEMWBS): development and UK validation.* Health and Quality of life Outcomes, 2007. **5**(1): p. 63.

For peer review only

40. Young, R.M. and R.G. Knight, *The Drinking Expectancy Questionnaire: A revised measure of alcohol-related beliefs.* Journal of Psychopathology and Behavioral assessment, 1989. **11**(1): p. 99-112.

41. McMahon, J. and B. Jones, *The negative alcohol expectancy questionnaire.* Journal of the Association of Nurses in Substance Abuse, 1993. **12**: p. 17.

42. The EuroQol Group, *EuroQol-a new facility for the measurement of health-related quality of life.* Health policy, 1990. **16**(3): p. 199-208.

43. Bray, J.W., et al., *Measuring economic outcomes of alcohol treatment using the Economic Form 90.* Journal of studies on alcohol and drugs, 2007. **68**(2): p. 248-255.

44. Eldridge, S.M., et al., *CONSORT 2010 statement: extension to randomised pilot and feasibility trials.* bmj, 2016. **355**: p. i5239.

45. Braun, V. and V. Clarke, *Using thematic analysis in psychology.* Qualitative research in psychology, 2006. **3**(2): p. 77-101.

46. May, C. and T. Finch, *Implementation, embedding, and integration: an outline of Normalization Process Theory.* . 2009: Sociology. p. 535-54.

47. May, C.R., et al., *Evaluating complex interventions and health technologies using normalization process theory: development of a simplified approach and web-enabled toolkit.* BMC health services research, 2011. **11**(1): p. 245.

48. May, C.R., et al., *Development of a theory of implementation and integration: Normalization Process Theory.* Implementation Science, 2009. **4**(1): p. 29.

49. Murray, E., et al., *Normalisation process theory: a framework for developing, evaluating and implementing complex interventions.* BMC medicine, 2010. **8**(1): p. 63.

For peer review only

Figure Legends

Figure 1: APPRAISE Logic Model

For peer review only

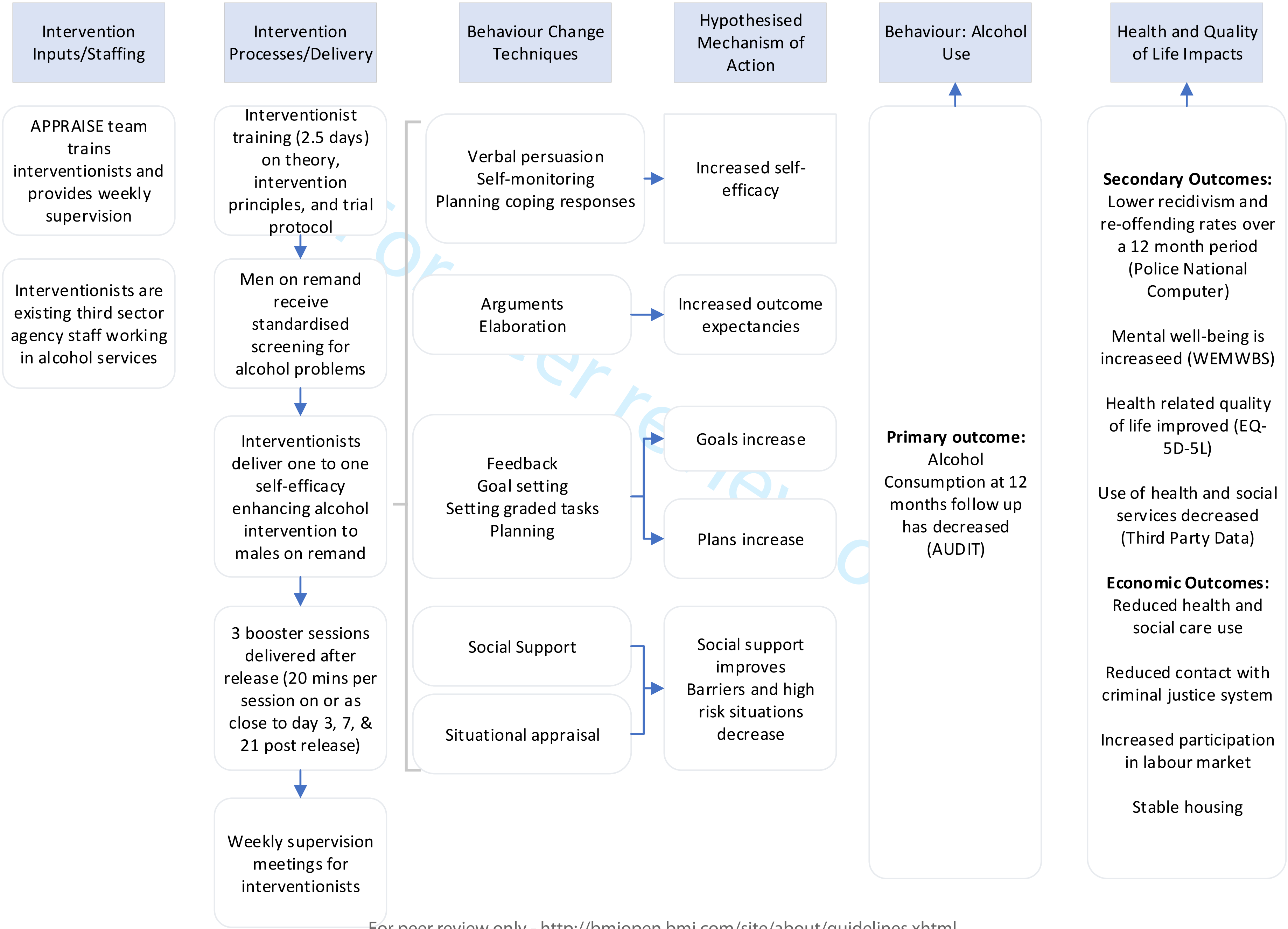
Intervention Inputs/Staffing
Intervention Processes/Delivery
Behaviour Change Techniques
Hypothesised Mechanism of Action
Behaviour: Alcohol Use
Health and Quality of Life Impacts
APPRAISE team trains interventionists and provides weekly supervision
Interventionists are existing third sector agency staff working in alcohol services
Interventionist training (2.5 days) on theory, intervention principles, and trial protocol
Men on remand receive standardised screening for alcohol problems
Interventionists deliver one to one self-efficacy enhancing alcohol intervention to males on remand
3 booster sessions delivered after release (20 mins per session on or as close to day 3, 7, & 21 post release)
Weekly supervision meetings for interventionists
Verbal persuasion
Self-monitoring
Planning coping responses
Arguments
Elaboration
Feedback
Goal setting
Setting graded tasks
Planning
Social Support
Situational appraisal
Increased self-efficacy
Increased outcome expectancies
Goals increase
Plans increase
Social support improves
Barriers and high risk situations decrease
Primary outcome: Alcohol Consumption at 12 months follow up has decreased (AUDIT)
Secondary Outcomes: Lower recidivism and re-offending rates over a 12 month period (Police National Computer)
Mental well-being is increaseed (WEMWBS)
Health related quality of life improved (EQ-5D-5L)
Use of health and social services decreased (Third Party Data)
Economic Outcomes: Reduced health and social care use
Reduced contact with criminal justice system
Increased participation in labour market
Stable housing
